# Cholesterol alters mitophagy by impairing optineurin recruitment and lysosomal clearance in Alzheimer’s disease

**DOI:** 10.1186/s13024-021-00435-6

**Published:** 2021-03-08

**Authors:** Vicente Roca-Agujetas, Elisabet Barbero-Camps, Cristina de Dios, Petar Podlesniy, Xenia Abadin, Albert Morales, Montserrat Marí, Ramon Trullàs, Anna Colell

**Affiliations:** 1 Department of Cell Death and Proliferation, Institut d’Investigacions Biomèdiques de Barcelona (IIBB), Consejo Superior de Investigaciones Científicas (CSIC), Institut d’Investigacions Biomèdiques August Pi i Sunyer (IDIBAPS), C/ Rosselló 161, 6th Floor, 08036 Barcelona, Spain; 2 Centro de Investigación Biomédica en Red sobre Enfermedades Neurodegenerativas (CIBERNED), Madrid, Spain; 3 Departament de Biomedicina, Facultat de Medicina, Universitat de Barcelona, Barcelona, Spain; 4 Neurobiology Unit, Institut d’Investigacions Biomèdiques de Barcelona (IIBB), Consejo Superior de Investigaciones Científicas (CSIC), Institut d’Investigacions Biomèdiques August Pi i Sunyer (IDIBAPS), Barcelona, Spain

**Keywords:** APP-PSEN1 mice, Mitochondria, Glutathione, Oxidative stress, PINK1, Parkin, Optineurin, Aggressomes

## Abstract

**Background:**

Emerging evidence indicates that impaired mitophagy-mediated clearance of defective mitochondria is a critical event in Alzheimer’s disease (AD) pathogenesis. Amyloid-beta (Aβ) metabolism and the microtubule-associated protein tau have been reported to regulate key components of the mitophagy machinery. However, the mechanisms that lead to mitophagy dysfunction in AD are not fully deciphered. We have previously shown that intraneuronal cholesterol accumulation can disrupt the autophagy flux, resulting in low Aβ clearance. In this study, we examine the impact of neuronal cholesterol changes on mitochondrial removal by autophagy.

**Methods:**

Regulation of PINK1-parkin-mediated mitophagy was investigated in conditions of acute (in vitro) and chronic (in vivo) high cholesterol loading using cholesterol-enriched SH-SY5Y cells, cultured primary neurons from transgenic mice overexpressing active SREBF2 (sterol regulatory element binding factor 2), and mice of increasing age that express the amyloid precursor protein with the familial Alzheimer Swedish mutation (Mo/HuAPP695swe) and mutant presenilin 1 (PS1-dE9) together with active SREBF2.

**Results:**

In cholesterol-enriched SH-SY5Y cells and cultured primary neurons, high intracellular cholesterol levels stimulated mitochondrial PINK1 accumulation and mitophagosomes formation triggered by Aβ while impairing lysosomal-mediated clearance. Antioxidant recovery of cholesterol-induced mitochondrial glutathione (GSH) depletion prevented mitophagosomes formation indicating mitochondrial ROS involvement. Interestingly, when brain cholesterol accumulated chronically in aged APP-PSEN1-SREBF2 mice the mitophagy flux was affected at the early steps of the pathway, with defective recruitment of the key autophagy receptor optineurin (OPTN). Sustained cholesterol-induced alterations in APP-PSEN1-SREBF2 mice promoted an age-dependent accumulation of OPTN into HDAC6-positive aggresomes, which disappeared after in vivo treatment with GSH ethyl ester (GSHee). The analyses in post-mortem brain tissues from individuals with AD confirmed these findings, showing OPTN in aggresome-like structures that correlated with high mitochondrial cholesterol levels in late AD stages.

**Conclusions:**

Our data demonstrate that accumulation of intracellular cholesterol reduces the clearance of defective mitochondria and suggest recovery of the cholesterol homeostasis and the mitochondrial scavenging of ROS as potential therapeutic targets for AD.

**Supplementary Information:**

The online version contains supplementary material available at 10.1186/s13024-021-00435-6.

## Background

Mitochondria are essential organelles critical to maintaining cellular homeostasis. Besides supplying the cell with ATP via oxidative phosphorylation (OXPHOS), mitochondria play a key role in the regulation of Ca^2+^ dynamics, redox signaling, and apoptosis [1]. Their function is even more important in neurons, with high bioenergetic demands and limited glycolytic capacity [2, 3]. Not surprisingly, mitochondrial dysfunction is an underlying common factor in many neurodegenerative processes, including Alzheimer’s disease (AD) [4–6].

Cells have different ways to cope with damaged and ROS-producing mitochondria. Depending on the severity and the type of damage, mitochondria can be repaired via intrinsic mitochondrial quality control mechanisms that include the action of mitochondrial proteases, the formation of mitochondrial-derived vesicles, and fission-fusion dynamics. Instead, when the insult persists they are eliminated by a mitochondrial-specific form of macroautophagy (hereafter referred as autophagy) called mitophagy that targets mitochondria for lysosomal degradation [7]. Mitophagy shares with autophagy the same machinery for initiation, expansion, and autophagosome engulfment, but requires specific proteins to recognize the damaged organelles [8, 9]. Different ubiquitin-dependent and independent pathways have been described, with specific mitochondrial receptors involved. Among them, the PTEN induced putative kinase 1 (PINK1)-parkin pathway is the most well-understood regarding its contributory role to neurodegenerative diseases, and particularly, to Parkinson’s disease (PD) [10, 11]. In healthy mitochondria, PINK1 is rapidly imported to the mitochondrial matrix where it is cleaved. The resulting fragments are released to the cytoplasm and degraded by the proteasome [8, 9]. Mitochondria depolarization, and also other injuries able to disrupt the mitochondrial protein import channel or impair the proteases involved in PINK1 processing, stabilize the full-length protein on the outer mitochondrial membrane (OMM). There, PINK1 through phosphorylating itself and different OMM proteins, including ubiquitin molecules, promotes the translocation and activation of the cytosolic E3 ubiquitin-protein ligase parkin, which initiates a multistep feed-forward process of phosphorylation and ubiquitination. The process culminates with the recruitment of ubiquitin-binding autophagy receptors, including nuclear dot protein 52 kDa (NDP52), sequestosome 1 (SQSTM1, also known as p62) and optineurin (OPTN). Via the LC3-interacting region (LIR) motif, all of them can bind the microtubule-associated protein 1 light chain 3 beta (MAP 1LC3B/LC3B)-positive phagophores, thereby priming mitochondria for autophagy [8, 9].

Recent studies have reported impaired mitophagy associated with the accumulation of dysfunctional mitochondria in hippocampal tissues from AD patients and AD iPSC-derived neurons [12, 13], suggesting a contributory role to AD progression. In line with this hypothesis, pharmacological and genetic manipulation strategies aimed at promoting mitophagy, via inducing the PINK1-parkin signaling axis, have been shown to significantly reduce Aβ depositions and prevent the cognitive impairment in AD mouse models [12–15]. Emerging experimental data indicate that microtubule-associated protein tau (MAPT/tau) and Aβ metabolism may interfere with the mitophagy machinery [16, 17]; however, the mechanisms that lead to mitophagy dysfunction in AD are still largely unknown. Recently, using a mouse model of AD that express the mutant amyloid precursor protein (Mo/HuAPP695swe) and presenilin 1 (PS1-dE9) together with the active sterol regulatory element-binding transcription factor 2 (SREBF2), we have shown that the increase of cholesterol burden in the brain disrupts key mechanisms of cellular clearance resulting in an abnormal build-up of autophagosomes and defective Aβ disposal [18, 19]. The intracellular rise of this sterol in neurons enhances autophagosomes formation by increasing the mitochondrial oxidative stress triggered by Aβ, but disrupts their fusion with the endosomal-lysosomal vesicles, and thereby the autophagy flux [18].. High cholesterol levels have been observed in vulnerable regions of post-mortem AD brains [20–22] and linked with enhanced Aβ production and mitochondrial oxidative stress [23]. In the current study, we address the role of cholesterol in mitophagy. Our results systematically illustrate how an increase in intracellular cholesterol content affects the PINK1-parkin pathway by regulating the mitochondrial antioxidant defense. Key differences are observed depending on whether changes in cholesterol levels are acute or persistent during AD progression, which should take into account in the design of therapeutic approaches that enhance PINK1-parkin-mediated mitophagy for the treatment of AD.

## Materials and methods

### Mice

Breeding pairs of B6C3-Tg (APPswe,PSEN1dE9)85Dbo/J (APP-PSEN1; MMRRC stock 34,829) [24] and B6;SJL-Tg (rPEPCKSREBF2)788Reh/J (SREBF2; JAX stock 003311) [25] mice were purchased from The Jackson Laboratory. APP-PSEN1 mice express a chimeric mouse/human amyloid precursor protein (isoform 695) with the Swedish mutation (Mo/HuAPP695swe) and mutant human presenilin 1 (PSEN1dE9), both directed to neurons by the mouse PRNP (prion protein) promoter. Both mutations are associated with early-onset Alzheimer disease. In turn, SREBF2 mice express a transgenic construct containing a human *SREBF2* cDNA fragment (amino acids 1–468) under the control of the rat PEPCK (phosphoenolpyruvate carboxykinase) promoter. APP-PSEN1-SREBF2 mice were generated from crossbreeding APP-PSEN1 and SREBF2 mice, which were back-crossed more than 10 generations into the B6SJL background and characterized as previously described [26]. Hippocampus from mice that overexpress SREBF2 show elevated mRNA expression levels of the LDL receptor and the cholesterol biosynthetic enzyme HMG-CoA synthase (Supplementary Figure 1) and accordingly, they display a 1.5-fold increase in total brain cholesterol levels and a 3-fold increase in mitochondrial cholesterol content compared to WT mice values [26]. No significant changes in cholesterol values are observed when comparing mice of different ages (4–10 months of age) [26]. At the time of weaning (21 d), mice were genetically identified by PCR using DNA from ear-tips and following the genotyping protocols provided by the supplier. Because APP-PSEN1-SREBF2 mice show sex-related differences in brain cholesterol levels, we only used male mice. In some cases, mitophagy was induced in vivo by intraperitoneal (i.p.) injection of rapamycin (5 mg/kg; Santa Cruz Biotech., sc-3504). Rapamycin was reconstituted in DMSO at 25 mg/ml and diluted in phosphate-buffered saline (PBS; Sigma-Aldrich, P3813) containing 5% Tween-80 (Sigma-Aldrich, P4780) and 5% PEG-400 (polyethylene glycol 400; Sigma-Aldrich, 202,398). In mice that overexpress SREBF2, the recovery of mitochondrial GSH content was accomplished by treatment with GSH ethyl ester (GSHee; 1.25 mmol/kg/day; Sigma-Aldrich, G1404), i.p. injected every 12 h for 2 weeks, as described [27].

### Human brain samples

Brain samples were supplied by the Biobank of Hospital Clínic, Barcelona - IDIBAPS (Barcelona, Spain). Clinical diagnosis and neuropathological changes were informed by the Biobank. The histological samples (5-μm sections) were obtained from the hippocampus of post-mortem brain of non-demented controls and AD patients classified according to the “ABC score” proposed by Montine et al. [28], which incorporates histopathologic assessments of amyloid β deposits (A, Thal Phase for Aβ plaques), staging of neurofibrillary tangles (B, Braak and BraakNFT stage), and scoring of neuritic plaques (C, CERAD neuriticplaque score). The age, gender, post-mortem interval and ABC score of the subjects are described in Table 1.

**Table 1:** Characteristics of human brain samples

“ABC” score	Gender	Age (yrs)	PMI (hh:mm)	Neuropathological changes
**Not or low AD**	M	70	16:30	Control
	M	76	11:30	AGD I
	M	66	7:00	Braak I, Thal 0, CERAD none
	F	68	13:00	Braak I-II, Thal 1, CERAD none
	F	70	5:00	Braak II, Thal 0, CERAD none, sparse hypoxia
**Interm. AD**	F	75	21:00	Braak III, Thal 3, CERAD moderate, SVD
	F	80	9:00	Braak III-IV, Thal 3, CERAD sparse
	M	73	4:20	Braak III, Thal 5, CERAD frequent, CAA sparse
	F	79	4:30	Braak IV, Thal 4, CERAD moderate, AGD III
	M	79	5:30	Braak IV, Thal 5, CERAD frequent, CAA sparse, SVD
**High AD**	M	79	6:25	Braak VI, Thal 4, CERAD frequent, CAA sparse
	F	76	10:00	Braak VI, Thal 5, CERAD frequent, CAA severe
	M	75	10:00	Braak VI, Thal 5, CERAD frequent, CAA sparse, LBD
	F	77	19:00	Braak VI, Thal 5, CERAD frequent, CAA sparse, HS
	F	74	14:30	Braak VI, Thal 5, CERAD frequent, CAA severe, LBD

Individuals are diagnosed according the “ABC” score, which incorporates histopathologic assessments of amyloid β deposits (A, Thal phase for Aβ plaques), the staging of neurofibrillary tangles (B, Braak and Braak NFT stage), and scoring of neuritic plaques (C, CERAD neuritic plaque score). Results are transformed into one of four levels of AD neuropathologic change: Not, Low, Intermediate, or High AD. *AGD* argyrophilic grain disease, *CAA* cerebral amyloid angiopathy, *HS* Hippocampal sclerosis, *LBD* Lewy bodies disease, *PMI* post-mortem interval, *SVD* small vessel disease

### Mitochondria isolation

Mitochondria were isolated from mouse brains as previously described in Yu et al. [29]. The method provides highly pure and structurally intact mitochondria, free from the endoplasmic reticulum, plasma membrane, and endosomal membrane contamination [27, 29]. In brief, brain was homogenized in mannitol buffer (210 mM Mannitol, 60 mM Sucrose, 10 mM KCl, 10 mM Succinate, 0.1 mM EGTA, 1 mM ADP, 0.25 mM DTT, and 10 mM HEPES, pH 7.4). For ubiquitin and phosphorylation analysis, EGTA was replaced by the cysteine protease inhibitor N-ethylmaleimide (10 mM) in the mannitol buffer. Homogenates were centrifuged at 700 g for 10 min, and the supernatants were centrifuged at 10,000 *g* for 15 min. The resulting pellet (the mitochondria-rich fraction) was suspended in 1 ml, loaded onto 8 ml of 30% (v/v) percoll gradient and centrifuged at 95,000 *g* for 30 min. The mitochondrial pellet was then rinsed twice by centrifuging 15 min at 10,000 *g*.

In SH-SY5Y cells, a mitochondria-rich fraction was obtained by digitonin permeabilization as described in Díaz et al. [30]. Briefly, we trypsinized and suspended 2.4 × 10^6^ cells in cold PBS supplemented with proteases inhibitors. We added 2 mg/ml digitonin previously boiled and incubated the samples on ice for 10 min. After the incubation period, the digitonin was immediately diluted with PBS and samples were centrifuged at 21,130 *g* for 5 min at 4 °C. The pellet was washed with PBS, centrifuged again, and proteins were extracted in RIPA buffer (Santa Cruz Biotech., sc-24,948).

### Cell cultures and treatments

Embryonic cortical-hippocampal neurons from mice were isolated at day 16–17 of pregnancy following a standard protocol. Dissociated cells were grown in Neurobasal™ medium (Thermo Fisher Sci., 21,103–049) supplemented with 2.5% (v:v) B27 supplement (Thermo Fisher Sci., 17,504–001), 0.5 mM L-glutamine (Sigma-Aldrich, G7513) and 5 μg/ml plasmocinTM (InvivoGen, ant-mpt), and plated onto poly-D-lysine (Sigma-Aldrich, P6407) and laminin (Sigma-Aldrich, L2020)-coated plates at a density of 2 × 10^5^ cells/cm^2^. Half of the culture medium was changed every 3 or 4 days. More than a 95% of neuronal purity was confirmed by immunochemistry using antibodies targeted to neuron and glial markers. Experiments were performed at 7 to 10 days in vitro (DIV). Cytotoxicity of the different compounds was assessed by lactate dehydrogenase (LDH) assay (Invitrogen™ CyQUANT™ LDH Cytotoxicity Assay, Thermo Fisher Sci., 16,280,972).

Human neuroblastoma-derived SH-SY5Y cells (ECACC Cat# 94030304, RRID:CVCL_0019) were grown in Gibco™ DMEM/F12, with GlutaMAX™ (Thermo Fisher Sci., 11,559,726) supplemented with 10% FBS (Thermo Fisher Sci., 11,550,356) and 5 μg/ml plasmocinTM (InvivoGen, ant-mpt).

Cholesterol enrichment of SH-SY5Y cells was achieved by using a cholesterol:methyl-β-cyclodextrin complex (CHO:MCD; Sigma-Aldrich, C4951). Cells were incubated with the complex (50 μg/ml of cholesterol) for 1 h, then washed with PBS, and incubated with new medium for 4 h. Cholesterol content was assessed by labeling the cells with 0.25 mg/ml filipin III (Sigma-Aldrich, F4767) for 20 min.

### Preparation of Aβ peptides

Human Aβ42 hydrochloride salt (Bachem, H-6466) was dissolved to 1 mM in hexafluoroisopropanol (HFIP; Sigma-Aldrich, 10,522–8) and aliquoted into microcentrifuge tubes, then the HFIP was evaporated and the peptides were stored at − 20 °C until use. For oligomeric assembly, concentrated peptides were resuspended by sonication in DMSO at 5 mM concentration and then diluted to 100 μM in phenol red-free medium and incubated at 4 °C for 24 h. Oligomeric forms of Aβ were confirmed by western blot, as previously described [26].

### Mt-mKeima mitophagy analysis

The coding sequence of the fluorescent mitochondrial-targeted monomeric Keima protein (mt-mKeima) [31] was cloned into the pWPI lentiviral vector (RRID:Addgene_12254; the plasmid was a gift from Didier Trono). The vector was packaged in lentiviral particles using psPAX2 (RRID:Addgene_12260; the plasmid was a gift from Didier Trono) and pMD2.G (RRID:Addgene_12259; the plasmid was a gift from Didier Trono). SH-SY5Y cells were transduced with a titration of the lentivirus containing the vector. The number of proviruses integrated per genome was assessed by determining the number of WPRE copies per diploid genome by digital polymerase chain reaction (dPCR). The number of diploid genomes was measured by multiplex amplification of two single copy nuclear genes: human TATA-box binding protein 1 (TBP1) and human mitochondrial transcription elongation factor (TEFM) as previously described [32] (primers sequences are detailed in Table 3). SH-SY5Y cells with 3 integrations/diploid genome were selected and collected by FACS (fluorescence-activated cell sorting). The cell sorter was programmed to collect only cell populations displaying low fluorescence intensity at Ex 488 nm and no signal at Ex 561 nm. Selected cells were grown and exposed to the indicated treatments. Dual-excitation ratio imaging of cells was carried out with two sequential excitation lasers (458 and 561 nm) in a Zeiss Lsm780 inverter laser scanning confocal microscope equipped with an argon laser, and using a 40x/1.2–0.28 water C-APO objective and a confocal pinhole set at 1 Airy unit. Images were taken on xyz axis with 6 slices per image. To obtain the mitophagy index, we followed the procedure described in Sun N. et al. [33] Briefly, we selected all pixels with a ratio of red/green pixels higher than 1.5 in a scatter plot (using the FIJI Image J software [34]) and the mitophagy index was calculated by dividing this number of pixels by the total of pixels after subtracting the background.

### Recombinant GST-PFO probe

The perfringolysin O (PFO) produced by *Clostridium perfringens* is a bacterial toxin that specifically binds cholesterol and forms pores. To evaluate the cholesterol content in human hippocampal tissues, we used a recombinant PFO fusioned with Glutathione-S-Transferase (GST-PFO), as described [35]. A pGEX4T plasmid vector containing the coding DNA sequence for PFO-GST, kindly provided by Dr. Kwiatkowska [35], was transformed into BL21(DE3) strain of *E. coli* (Thermo Fisher Sci., EC0114). Bacteria were grown at 37 °C in LB medium containing 100 μg/ml ampicillin to OD = 0.6, when 0.5 mM IPTG was added. After 20 h at 18 °C, bacteria were centrifuged at 4000 *g* for 10 min at 4 °C, washed with PBS and incubated 10 min at 4 °C in lysis buffer (50 mM Tris pH 8, 100 mM NaCl, 1 mM EGTA, 1 mM EDTA, 5 mM β-mercaptoethanol, 1 mM PMSF, 0.35 mg/ml lysozyme and proteases inhibitor cocktail). The suspension was sonicated on ice for 15 min at 0.3 cycles and amplitude 33% after adding 1% Triton X-100. The lysate was clarified at 18,000 *g* for 30 min at 4 °C and loaded onto a GSTrap™ 4B column (GE Healthcare, 28–4017-45). Purification was made according to the commercial protocol, adding 5 mM DTT and 1% Triton X-100 to the wash buffer. GST-PFO was eluted with 10 mM GST, 5 mM DTT, 50 mM Tris pH 8 and the elute was filtered using Amicon Ultra-15 10 k centrifugal filters (Merk, UFC901008). The protein was aliquoted and stored at − 80 °C with 20% sucrose. Purity of recombinant GST-PFO was assessed by western blot in comparison with the unbound proteins of the lysate.

### Protein-lipid overlay assay

To analyze the ability of the recombinant GST-PFO to recognize cholesterol, we performed a protein-lipid overlay assay, as described [35]. 1 μl of the CHO:MCD complex solution containing 100–800 pmols of cholesterol was spotted onto a 0.2 μm nitrocellulose membrane (GE Healthcare, 1,060,015). Once completely dry, the membrane was pressed with a hot block at 60 °C for 5 s to fix the lipid. Then, it was blocked with 5% nonfat milk for 1 h and incubated with 2 μg/ml recombinant GST-PFO in TBS with 0.1% Tween-20 (TBS-T) for 1 h at RT. Protein-lipid blots were probed with anti-GST (1:1.000; Santa Cruz Biotech., sc-374,171) overnight at 4 °C, washed three times and probed with mouse HRP-linked IgG antibody (1:20,000; Sigma-Aldrich, GENA931) in TBS-T with 1% nonfat milk for 1 h at RT. Bound antibodies were revealed using Clarity Max ECL western blotting substrate (Bio-Rad, 1,705,062).

### Western blot analysis

**Fig. 1:**
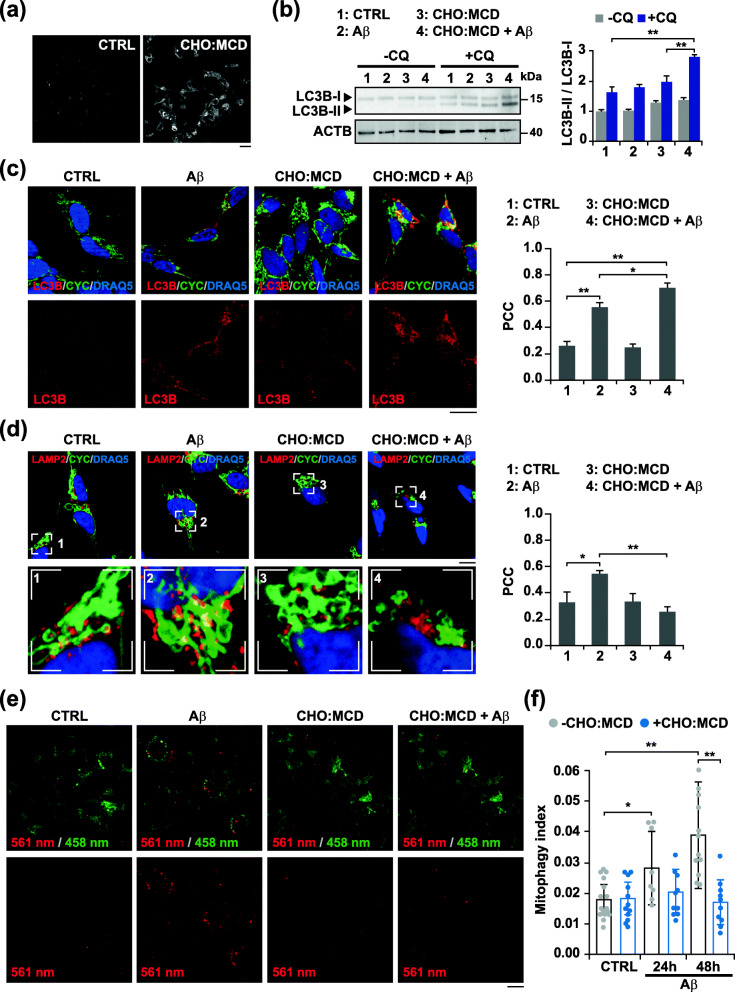
Cholesterol-enrichment in SH-SY5Y cells enhances Aβ-induced mitophagosomes formation while impairing mitochondrial clearance by lysosomes. Cells were incubated with a complex of cholesterol:methyl-β-cyclodextrin (CHO:MCD) containing 50 μg/ml cholesterol during 1 h followed by 4 h of recovery. **a** Filipin staining of unesterified cholesterol. Representative images of the increased fluorescence after CHO:MCD treatment (*n* = 3). Scale bar: 25 μm. **b** Representative immunoblots of cell extracts showing LC3B levels. To assess changes in the autophagy flux, cells were treated with chloroquine (CQ, 10 μM). Densitometry values of the bands were normalized to values of the corresponding ACTB/actin β bands and expressed as LC3B-II/LC3B-I ratio (*n* = 3). See Supplementary Figure 16 for uncropped blots. **c** and **d** Representative confocal images of control (CTRL) and cholesterol-enriched cells after treatment with Aβ (10 μM) for 24 h, and double immunostained with antibodies for LC3B (red) and CYC (green) (**c**) and for LAMP2 (red) and CYC (green) (**d**). The Pearson’s correlation coefficient (PCC) was calculated from 3 independent experiments (at least 6 random fields were analyzed per condition). Nuclei were counterstained with DRAQ5 (blue). Insets show a 3-fold magnification of the indicated regions. Scale bar: 25 μm. **e** Dual-excitation ratiometric imaging of mt-mKeima. Representative confocal images of control and cholesterol-enriched cells stably expressing mt-mKeima after Aβ (10 μM) incubation for 48 h. The emission signal obtained after excitation with the 458 nm laser is shown in green, and that obtained after excitation with the 561 nm laser is shown in red. Scale bar: 25 μm. **f** Mitophagy index calculated from 3 independent experiments (at least 10 random fields were analyzed per condition). One-way ANOVA. *P < 0.05; **P < 0.01 (data are mean ± SD)

Lysates were prepared in RIPA buffer (Santa Cruz Biotech., sc-24,948). Samples (20 to 70 μg of protein/lane) were resolved by SDS-PAGE and transferred to nitrocellulose membranes. Protein transfer was checked by Ponceau S solution (Sigma-Aldrich, P7170). Blots were probed with the primary antibodies listed in Table 2 overnight at 4 °C. The membranes were then incubated with anti-rabbit (1:10,000), anti-mouse (1:20,000) or anti-rat (1:40,000) IgG horseradish peroxidase-coupled secondary antibodies (Sigma-Aldrich, A8275, GENA931, GENA935, respectively) and the immunoreactive bands were detected using Clarity ECL western blotting substrate (Bio-Rad, 1,705,061) or Clarity Max ECL western blotting substrate (Bio-Rad, 1,705,062) for low protein concentrations. To analyze phosphorylated proteins, isolated mitochondria were subjected to Phos-tag SDS-PAGE. Resolving gels were made with 7% acrylamide, 100 nM MnCl_2_ and 50 μM Phos-tag™ AAL-107 (Wako Chemicals, 304–93,521), according to manufacturer instructions. After electrophoresis, Phos-tag gels were soaked 2 times in transfer buffer (25 mM Tris-HCl pH 8.3, 192 mM glycine, 0.05% SDS, and 20% methanol) containing 1 mM EDTA for 10 min, with gentle agitation for the elimination of the manganese ions from the gel. The resolved proteins were transferred to PVDF membranes previously activated 30 s in 100% methanol and equilibrated 15 min in transfer buffer. Blots were probed with the primary antibodies detailed in Table 2. Uncropped scans of representative western blots from Figs. 1, 3, 4 and 5 are shown in Supplementary Figures 16 to 19.

**Table 2:** Details of the primary antibodies used in the study

Antibody	Company	Cat. Number; RRID	WB dilution	IF/IHC dilution
ACTB	Sigma-Aldrich	A3854; RRID:AB_262011	1:40,000	
CTSD	Santa Cruz Biotech.	sc-6486; RRID:AB_637896	1:500	
CYC	BD Biosciences	556,433; RRID:AB_396417	1:1000	
CYC	BD Biosciences	556,432; RRID:AB_396416		1:300
GAPDH	Abcam	ab8245; RRID:AB_2107448	1:5000	
GST	Santa Cruz Biotech.	sc-374,171; RRID:AB_11008206	1:1000	1:300
HDAC6	Santa Cruz Biotech.	sc-5258; RRID:AB_2116625		1:300
LAMP2	Abcam	ab13524; RRID:AB_2134736		1:300
LC3B	Cell Signaling	#2775S; RRID:AB_915950	1:1000	1:300
MFN2	Santa Cruz Biotech.	sc-50,331; RRID:AB_2142754	1:500	
OPTN	Santa Cruz Biotech.	sc-166,576; RRID:AB_2156554	1:1000	1:300
SQSTM1	Abcam	ab91526; RRID:AB_2050336	1:1000	1:500
PARL	Aviva Systems Biology	ARP44851_P050; RRID:AB_1294596	1:1000	
Parkin	Abcam	Ab77924; RRID:AB_1566559	1:1000	1:1000
PDH	Abcam	ab110330; RRID:AB_10858459	1:1000	
PGC-1α	Millipore	ST1202; RRID:AB_2237237	1:1000	
PINK1	Abcam	ab23707; RRID:AB_447627	1:1000	1:500
PINK1	Novus Biotech.	BC100–494; RRID:AB_10127658	1:1000	
RHOT1	Biorbyt	orb165560	1:1000	
p-TBK1 (Ser172)	Cell Signaling	#5483; RRID:AB_10693472	1:1000	
TBK1	GeneTex	GTX113057; RRID:AB_11174793	1:1000	
TFAM	GeneTex	GTX103231; RRID:AB_11176720	1:1000	
TOMM20	Santa Cruz Biotech.	sc-11,415; RRID:AB_2207533	1:500	1:300
Ub	Santa Cruz Biotech.	sc-9133; RRID:AB_2180553	1:500	
K63-Ub	Affymetrix	14–6077	1:500	1:300
ULK1	Santa Cruz Biotech.	sc-33,182; RRID:AB_2214706	1:250	

### Immunofluorescence and laser confocal imaging

Paraffin-embedded blocks from mouse brains were prepared by sequential dehydration in graded ethanol and infiltration in paraffin before embedding. Blocks were serially sectioned between − 1.2 mm and − 2.4 mm from Bregma at a thickness of 5 μm. Dewaxed and rehydrated hippocampal sections from mouse and human brains were first boiled in Tris-EDTA buffer, pH 9.3. Then, sections were blocked in Antibody Diluent with Background Reducing Components (Dako, S3022) for 20 min at RT and incubated overnight at 4 °C with the antibodies listed in Table 2. After washing with PBS, the immunoreaction was visualized using the following secondary antibodies: anti-goat Cy3 (1:300, Jackson ImmunoResearch Labs Cat# 705–166-147, RRID:AB_2340413), anti-rabbit Cy3 (1:300, Jackson ImmunoResearch Labs Cat# 711–166-152, RRID:AB_2313568), anti-rabbit Alexa Fluor 488 (1:300, Molecular Probes Cat# A-21206, RRID:AB_2535792), anti-mouse Alexa Fluor 488 (1:300, Molecular Probes Cat# A-21202, RRID:AB_141607) or anti-mouse Alexa Fluor 555 (1:300, Thermo Fisher Scientific Cat# A-31570, RRID:AB_2536180). Nuclei were stained with DRAQ5 fluorescent probe solution (5 μM; Biostatus, DR50200) or bisBenzimide Hoechst 33258 (10 μg/ml; Sigma Aldrich; B2883). Hippocampal-cortical neurons and SH-SY5Y cells were fixed with 4% paraformaldehyde and permeabilized with 0.1% saponin (Sigma-Aldrich, 47,036) in blocking buffer (1% BSA and 0.5% glycine in PBS) for 15 min before adding the primary antibodies. In some cases, samples were incubated with 20 μg/ml GST-PFO in antibody diluent (Antibody Diluent with Background Reducing Components) for 3 h (tissue sections) or 45 min (cells), and washed three times with PBS before proceeding with the immunostaining. Fluorescence microphotographs were acquired with a Nikon Eclipse E-100 microscope using the objective 40x/0.75 plan fluor DLL. Confocal images were collected with a Leica TCS SPE laser scanning confocal microscope using the objectives 20x/0.7 HC PL APO and 63x/1.4–0.60 oil HCX PL APO, and a confocal pinhole set at 1 Airy unit. For whole hippocampus imaging, individual images were acquired and stitched using a 20x/0.17 MI-oil plan fluor objective in an Andor Dragonfly spinning disk confocal microscope equipped with a Zyla 4.2 PLUS sCMOS camera and coupled with Fusion software (Andor).

### Mitochondrial DNA (mtDNA) quantification

The prefrontal cortex, hippocampus and cerebellum from mouse brains were added directly into 800 μl of the solubilization reagent DireCtQuant 100ST (Frontex Biomed, #DCQ100ST) and homogenized in tubes with glass beads at 350 rpm for 45 s. After homogenization, each sample was incubated at 90 °C for 3 min, cooled to RT and centrifuged at 10,000 *g* for 10 min. The supernatant was diluted 1:3 in DireCtQuant 100ST and stored at − 20 °C. Prior quantification, the samples were diluted to 1:16 and 1:16,000 for genomic and mitochondrial measurements, respectively. Copies of mtDNA were quantified by dPCR. Before dPCR, we set up a restriction enzyme digestion as described in Podlesniy and Trullas [36]. Briefly, we added 8.8 μl of the diluted sample into a new tube with 0.5 μl of both SaqA1 and BsuRI (for genomic DNA) or 1 μl FastDigest SspI (for mtDNA) restriction enzymes, 10 μl of 2X QX200 ddPCR EvaGreen Supermix (Bio-Rad, 186–4033) and the corresponding primer mix (Table 3) to a final PCR volume of 20 μl. To quantify the number of genome copies we used two sets of primers targeting two different single-copy nuclear genes that produce amplicons of different sizes (*Bax*-72 and *Gsk3β*-86). The reaction mixture was incubated for 15 min at 37 °C. After restriction digestion, the mixture was partitioned and emulsified in 65 μl of droplet generation oil for EvaGreen (Bio-Rad, 186–4005) in a QX200 Droplet Generator. The emulsion was transferred to a 96-wells plate and dPCR was performed in a C1000 Touch Thermal cycler (Bio-Rad) with the following cycling conditions: 95 °C for 5 min, 95 °C for 30 s and 57 °C (for mtDNA) or 60 °C (for genomic DNA) for 1 min for 40 cycles, 4 °C for 5 min, 90 °C for 5 min and 11 °C for storage before dPCR analysis. The quantification of positive and negative droplets was assessed using a QX200 Droplet Reader. Results were analyzed with QuantaSoft™ Analysis Pro software. Based on the ratio of positive to total droplets, the software estimates the concentration of target molecules per reaction, which was used to calculate the absolute number of copies of the target molecules per volume of sample analyzed. To calculate the numbers of gene copies per diploid genome, the number of copies of mtDNA was divided by the number of copies of the single-copy genes *Bax* and *Gsk3β* present in the sample.

**Table 3:** Primer sequences used for selfie qRT-PCR and dPCR analysis

Amplicon	Forward sequence	Reverse sequence
*Bax*-72	CACTGCCTTGGACTGTGTCT	CCTTTCCCCTTCCCCCATTC
*Gsk3β*-81	CGAACTCCACCAGAGGCAAT	AGCTTCCAGTGGTGTTAGCC
*Hmgcs1*	TGCACGGATCGTGAAGACAT	GCCGCCCAATGCAATCATAG
*Ldlr*	GTGAGACAACAGCCCTCCTC	CAGGTGAATTTGGGCGAGTG
*mtDNA-80*	AGCTCAATCTGCTTACGCCA	GCAATAACAAGTGCTATGTGGCT
*TBP1*–73	CACCACAGCTCTTCCACTCA	GGGGAGGGATACAGTGGAGT
*TEFM*-88	GTGACTCCCGGACTAGTGGA	GATGGGAAGAACACCCGAGG
*Tfam*	GGGAATGTGGAGCGTGCTAA	GATAGACGAGGGGATGCGAC
*WPRE-106*	TGGACAGGGGCTCGGCTGTT	CGCGCAGAATCCAGGTGGCA

### Selfie RT-qPCR

We determined the mRNA expression levels of *Tfam, Ldlr and Hmgcs1* by Selfie RT-qPCR, a method that determines the absolute amount of an RNA transcript produced by its coding DNA by measuring, in the same sample containing unpurified RNA and DNA, the amount of DNA amplified by qPCR before and after reverse transcription [36]. Briefly, the hippocampus isolated from mice brains was homogenized, and the resulting samples were diluted as described in the above section. We performed a pre-annealing step in duplicate, mixing the lysed sample with 0.5 μM reverse primer (complementary to *Tfam* RNA transcript) at 70 °C for 5 min. Next, we added RiboLock RNase Inhibitor (ThermoFisher Sci., EO0381), 10 mM dNTPs and glycerol or Maxima H Minus Reverse Transcriptase (ThermoFisher Sci., EP0751), and the mixture was retro-transcribed for 30 min at 60 °C and 5 min at 85 °C. Finally, 0.5 μM forward primer was added and amplified by conventional RT-qPCR (5 min at 95 °C, followed by 40 cycles of 15 s at 95 °C, 35 s at 60 °C and 25 s at 72 °C), using SsoAdvanced™ Universal SYBR® Green Supermix (Bio-Rad, 1,725,272). The number of transcripts per encoding gene present in the sample was calculated by subtracting the amount obtained before reverse transcription from that obtained after reverse transcription yields and divided by the amount obtained before reverse transcription.

### Endo-lysosome and autophagosome fractionation

Autophagosomes and endo-lysosomes were isolated by centrifugation in a discontinuous nycodenz gradient, as described previously [37]. The corresponding membrane fraction resulting from 5 brains was suspended in 1 ml of 50% nycodenz and loaded onto 3.5 ml of 26% nycodenz placed at the bottom of the tubes. Density gradient separation of autophagic vacuoles was achieved after layering above the sample with 1.5 ml of each 24, 20 and 10% nycondenz, and ultracentrifugation at 26,000 rpm for 4 h in a SW40Ti rotor (Beckman Coulter). The bands were collected from the gradient, diluted five times with 0.3 M sucrose and sedimented at 37,000 g for 10 min. Autophagosomes were recovered in the 20–15% interface and endo-lysosomes in the 26–24% interface. Purity and efficiency was evaluated as described previously [18]. Autophagosomal and lysosomal fractions were both individually labeled with anti-LC3 antibody plus FITC-labeled secondary antibody and LysoTracker Red. The number of particles for each fluorophore was quantified and divided by the total count from its corresponding phase contrast images (Supplementary Figure 2). We only used fractions with a percentage of positive stained particles per microscopic field over 70%.

### Statistics

Results are expressed as means ± standard deviation (SD) of the number of experiments. Statistical comparisons were performed using unpaired 2-tailed Student’s t-test or one-way analysis of variance followed by Tukey’s test for multiple comparisons. Pearson’s correlation coefficient (PCC) was determined as a statistic for quantifying colocalization using the FIJI ImageJ plugin Coloc 2 with a ROI selection of interest in some cases [34]. A *P* < 0.05 value was considered statistically significant.

## Results

### Cholesterol enhances mitophagosome formation evoked by Aβ while impairing mitochondrial clearance by lysosomes in SH-SY5Y cells

To study the effect of intracellular cholesterol on mitophagy, we first used the human neuroblastoma SH-SY5Y cells treated with a water-soluble cholesterol complex (CHO:MCD, cholesterol:methyl-β-cyclodextrin) and exposed to 10 μM oligomeric Aβ for 24 h. The increase of cholesterol content after treatment was confirmed by staining the cells with filipin (0.25 mg/ml), a fluorescent polyene antibiotic that specifically binds cholesterol. Fluorescence microscopy images showed a higher and homogeneous intracellular staining in CHO:MCD-treated cells (Fig. 1a). We had previously shown that cholesterol loading using this water-soluble complex resulted in a 3 times increase of mitochondrial cholesterol levels associated with a nearly 60% decrease of the mitochondrial GSH content [19]. Upon autophagy induction, a key requirement for autophagosome formation is the recruitment of cytosolic LC3B to the phagophore membranes and the subsequent conjugation to phosphatidylethanolamine. Therefore, levels of lipidated LC3B (also known as LC3B-II) have been widely used as a measure of autophagosome content. As shown, Aβ exposure induced autophagosome synthesis, with a significant increase of the LC3B-II:LC3B-I ratio in cholesterol-enriched cells when the autophagy flux was blocked with the lysosomotropic reagent chloroquine (CQ) (Fig. 1b). To analyze mitophagy in cells, we next performed double immunostaining with antibodies against LC3B and cytochrome C (CYC), used as a marker of mitochondria (Fig. 1c). As expected, confocal microscopy showed a greater presence of autophagosomes (LC3B puncta) after Aβ incubation. The degree of colocalization between LC3B-positive vesicles and the mitochondrial marker was higher in cholesterol-enriched cells (Fig. 1c). However, despite the enhanced formation of autophagosomes, no overt co-localization between the lysosomal marker LAMP2 (lysosomal associated membrane protein 2) and CYC was observed when the cellular cholesterol content was high (Fig. 1d), therefore, suggesting that mitophagosomes accumulation in cholesterol-enriched cells is in part due to defective autophagy resolution.

To more readily assess the mitophagy flux in living cells, we used the monomeric Keima probe targeted to the mitochondrial matrix with a mitochondria targeting signal peptide sequence (mt-mKeima) [31]. mKeima is a pH-sensitive, dual-excitation ratiometric fluorescent protein that also exhibits resistance to lysosomal proteases. At the physiological pH of the mitochondria the excitation of mt-mKeima at 440 nm predominates, however, when mitochondria are delivered to the acidic lysosomal environment, the peak of the excitation spectrum of mt-Keima shifts from 440 to 586 nm. Taking advantage of these properties, lentiviral-transduced SH-SY5Y cells stably expressing mt-mKeima were cholesterol-enriched and the formation of autolysosomes was analyzed by confocal microscopy after Aβ exposure (Fig. 1e). In control cells exposed to the cytotoxic peptide up to 48 h, the dual-excitation ratiometric imaging of mt-mKeima showed cytoplasmic puncta structures with strong signals at an excitation wavelength of 561 nm (Fig. 1e), which were already evident after 24 h of Aβ incubation (Supplementary Figure 3). Interestingly, and consistent with the results obtained by immunocytochemistry, no fluorescence signal was observed at 561 nm when the cells exposed to Aβ were previously enriched with cholesterol, indicating that mitochondrial delivery to lysosomes was disrupted (Fig. 1e and Supplementary Figure 3). Accordingly, the mitophagy index calculated from these ratiometric images, as previously described [33], increased cumulatively over time in control cells exposed to Aβ while remained unchanged in CHO:MCD-treated cells (Fig. 1f). Similar results were observed when mitophagy was triggered by the mitochondrial uncoupler carbonyl cyanide chlorophenylhydrazone (CCCP) (Supplementary Figure 4), thus further proving that rise of intracellular cholesterol levels impairs mitophagy flux regardless of mitochondrial insult.

### Cholesterol-mediated depletion of mitochondrial GSH promotes incomplete PINK1-mediated mitophagy in embryonic cortical/hippocampal neurons exposed to Aβ

**Fig. 2:**
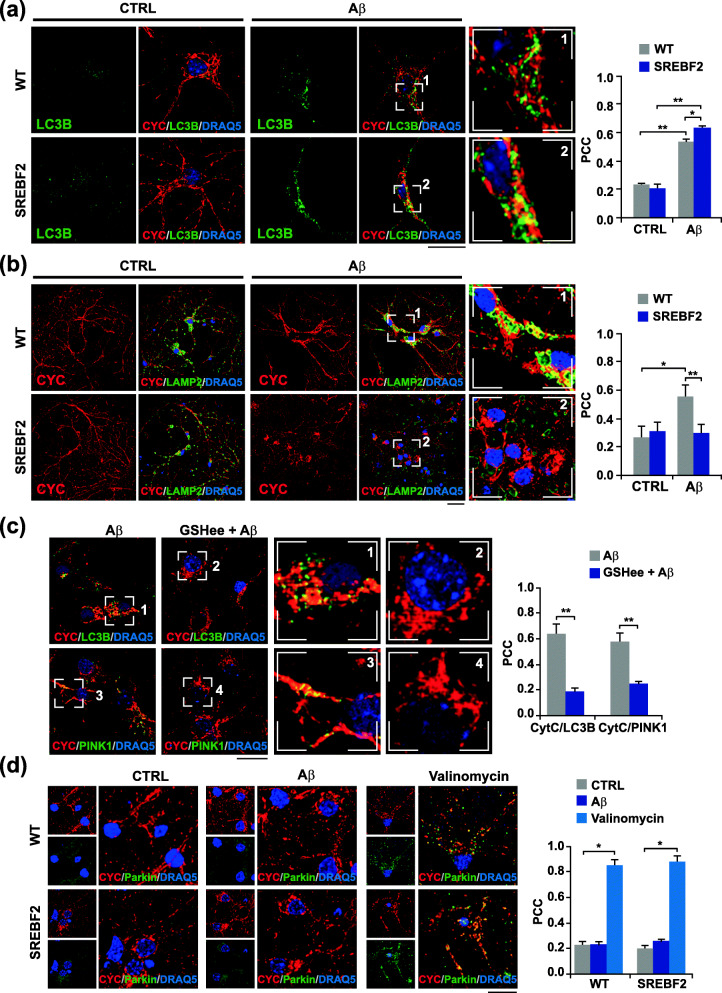
Cholesterol-induced depletion of mitochondrial GSH stimulates incomplete PINK1-mediated mitophagy in cultured primary neurons exposed to Aβ. Embryonic cortical and hippocampal neurons isolated from WT and SREBF2 mice were treated with Aβ (5 μM) for 24 h or valinomycin (10 μM) for 3 h to trigger mitophagy. **a** and **b** Representative confocal images of neuronal-enriched cultures double immunostained with antibodies for LC3B (green) and CYC (red) (**a**) and for LAMP2 (green) and CYC (red) (**b**). Scale bar: 25 μm. **c** SREBF2 cells were pre-incubated with GSH ethyl ester (GSHee, 0.5 mM) for 30 min prior mitophagy induction with Aβ. Shown are representative confocal images of double immunostainings for LC3B (green) and CYC (red) and for PINK1(green) and CYC (red). Scale bar: 25 μm. **d** Representative confocal images of a double immunolabeling for CYC (red) and parkin (green). Scale bar: 25 μm. Nuclei were counterstained with DRAQ5 (blue). Insets show a 3-fold magnification of the indicated regions. In all the cases, the Pearson’s correlation coefficient (PCC) was calculated from 3 independent experiments (at least 6 random fields were analyzed per condition). *P < 0.05; **P < 0.01 (data are mean ± SD)

Most of the studies regarding mitophagy regulation have been performed in transformed cell lines using the mitochondrial uncoupler CCCP as a trigger. Remarkably, CCCP-induced mitophagy is significantly attenuated when cells are forced to depend on mitochondrial respiration [38]. Because of the high energetic dependence of neurons on mitochondrial function [3], and the unability of neurons to dilute mitochondrial damage through cell division, it is possible that the mitophagy changes observed in cholesterol-enriched SH-SHY5Y cells may be different in primary neuronal cultures. To evaluate this possibility, embryonic cortical/hippocampal neurons from wild-type (WT) and SREBF2 mice were incubated with Aβ at 5 μM for 24 h and double immunostained with anti-LC3B and anti-CYC antibodies (Fig. 2a). Cell viability remained unaffected at the length of the incubation period and doses used (Supplementary Figure 5). Confocal images revealed a marked accumulation of LC3B-positive structures in the soma of the cells after Aβ treatment, which partially co-localized with the mitochondrial marker (Fig. 2a). The degree of mitochondria co-localization with LC3B puncta was significantly higher in SREBF2 cells compared to WT cells (Fig. 2a). Similarly, an enhanced presence of mitochondria in autophagosomes was observed in SREBF2 cells when we used a common autophagy inducer like the mTOR inhibitor rapamycin (Supplementary Figure 6a) at sub-toxic concentrations (Supplementary Figure 5), thereby confirming that changes in cholesterol homeostasis in cultured primary neurons regulate mitophagy to the same extent as in transformed cell lines. In parallel, mitophagy resolution was assessed by co-immunostaining with anti-LAMP-2 and anti-CYC antibodies. Confocal microscopy showed an increased amount of double-stained CYC- and LAMP-2-positive structures in WT cells elicited by Aβ (Fig. 2b) and rapamycin (Supplementary Figure 6b). In contrast, no evident co-localization was found between mitochondria and the lysosomal marker in SREBF2 cells (Fig. 2b and Supplementary Figure 6b), hence, recapitulating the mitophagy flux defect observed in cholesterol-enriched SH-SY5Y cells. It is also noteworthy the difference in organelle distribution observed after mitophagy engagement. Whereas in WT cells, lysosomes were mainly confined in the neuronal soma and co-localized with the mitochondrial marker, in SERBF2 cells the lysosomes remained evenly distributed in the perikaryon and along neurites after Aβ or rapamycin insult (Fig. 2b and Supplementary Figure 6b). Furthermore, unlike WT cells, mitochondria in SERBF2 cells mostly accumulated in the soma once mitophagy was induced, displaying a more fragmented pattern in neurites (Fig. 2b and Supplementary Figure 6b). In agreement with these findings, a similar abnormal distribution was previously reported in pyramidal neurons of AD-affected individuals [39].

Early steps of the nascent autophagosome synthesis require a transient generation of mitochondrial ROS [40]. As previously mentioned, we have recently demonstrated that an increase in intracellular cholesterol levels provides the necessary local burst of ROS to trigger autophagosomes formation by reducing the mitochondrial GSH levels and favoring mitochondrial Aβ-induced oxidative stress [18]. Furthermore, the presence of LC3B-positive vesicles elicited by Aβ in cholesterol-enriched neuronal cultures is abolished after treatment with GSH ethyl ester (GSHee), a cell-permeable form of GSH that recovers the pool of mitochondrial GSH and prevents Aβ-induced oxidative damage [18]. Consistent with these data, pretreatment with GSHee significantly inhibited mitophagy in SREBF2 cells (Fig. 2c). After mitochondrial GSH recovery, confocal photomicrographs of SREBF2 cells exposed to Aβ displayed a marked decrease of CYC- and LC3B-positive vesicles. The treatment with GSHee also reduced the mitochondrial accumulation of PINK1 in these cells, with confocal photomicrographs showing a loss of co-localization between the key mitophagy player and the mitochondrial marker (Fig. 2c). Interestingly, Aβ promoted PINK1 presence in mitochondria from SREBF2 cells without loss of mitochondrial membrane potential, assessed by tetramethyl rhodamine methyl ester (TMRM), a cell-permeant fluorescent dye that only accumulates in mitochondria with intact membrane potential (Supplementary Figure 7). Moreover, confocal microscopy analyses revealed that mitochondrial stabilization of PINK1 and the subsequent mitophagy induced by Aβ in SREBF2 cells occurred without parkin recruitment (Fig. 2d). Translocation of the cytosolic E3 ubiquitin-protein ligase was only observed when cells were treated with the potassium ionophore valinomycin, as shown by the punctate parkin-positive staining that highly co-localizes with the mitochondrial marker (Fig. 2d). Altogether, these findings indicate that Aβ triggers a PINK1-mediated but parkin-independent mitophagy in cultured primary neurons, which is exacerbated by cholesterol-induced depletion of mitochondrial GSH and does not require the loss of mitochondrial membrane potential.

### SREBF2 overexpression in APP-PSEN1 neurons induces PINK1-mediated accumulation of mitophagosomes but prevents mitophagy completion

To further explore the impact of cholesterol on the mitophagy pathway during AD progression, we used both primary neuronal cultures and brains from WT and APP-PSEN1 mice with and without overexpressing SREBF2, at different ages. Of note, unlike the SREBF1 isoform, a strong activator of lipogenic genes, SREBF2 mainly induces cholesterogenic genes [41]; accordingly, SREBF2-overexpressing mice display enhanced levels of cholesterol in all the tissues analyzed, while triglyceride content, although slightly elevated in the liver, remain unchanged in the plasma and peripheral tissues [25].

In neuronal cultures, consistent with the reported blockage of the last steps of autophagy by high cholesterol burden, no colocalization was found between mitochondria and the lysosomal marker LAMP2 in APP-PSEN1-SREBF2 cells despite mitophagosomes formation (Supplementary Figure 8a). Confocal analyses also revealed an increased presence of PINK1 and the autophagy receptor SQSTM1 in mitochondria of APP-PSEN1-SREBF2 cells (Supplementary Figure 8b). However, as in SREBF2 cells exposed to Aβ, the mitochondrial accumulation of PINK1 did not stimulate the recruitment of parkin (Supplementary Figure 8c). Translocation of the ubiquitin ligase to mitochondria occurred only when cells were treated with valinomycin, which significantly reduced the mitochondrial membrane potential as expected (Supplementary Figure 8d).

### APP-PSEN1-SREBF2 brains show mitophagy failure with disrupted mitochondrial recruitment of the mitophagosome synthesis machinery despite high PINK1/parkin mitochondrial presence

**Fig. 3:**
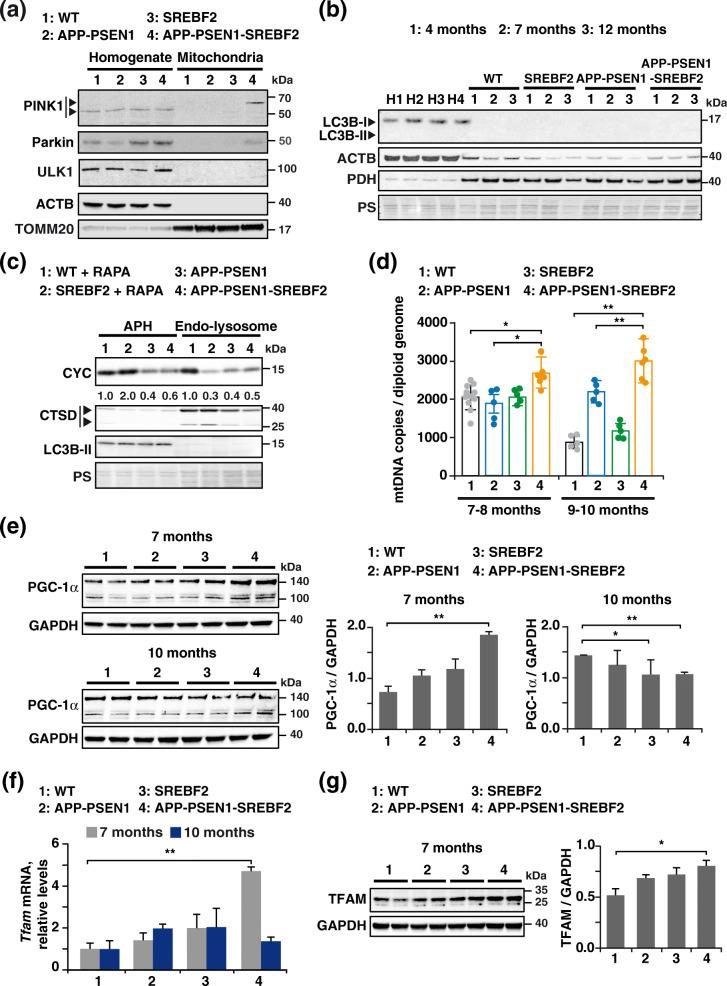
APP-PSEN1-SREBF2 brains show mitophagy impairment with disrupted recruitment of core proteins for mitophagosome synthesis that leads to mitochondria accumulation. **a** Western blot analysis of PINK1, parkin, and ULK1 in homogenates and the mitochondrial fraction of brains from 8-month-old WT (wild-type) and the indicated transgenic mice. Arrows in the PINK1 blot indicate the mature (52-kDa) and full-length (62-kDa) form. ACTB/actin β and TOMM20 levels were used as protein loading control in homogenates and mitochondria samples, respectively. **b** Immunoblot analysis of LC3B levels in mitochondria isolated from brains of WT and the indicated transgenic mice from 4 to 12 months of age. H1-H4: homogenate from 9-month-old WT (1), SREBF2 (2), APP-PSEN1 (3), and APP-PSEN1-SREBF2 (4) mice. **c** Western blot analysis of CYC in autophagosome (APH) and endo-lysosomes isolated from brains of 8-month-old WT and the indicated transgenic mice. To induce mitophagy, WT and SREBF2 mice were treated with rapamycin (RAPA, 5 mg/kg, i.p.) for 24 h. All densitometry values were first normalized to Ponceau S (PS) staining to adjust for protein loading. Then, values of CYC bands were normalized to the values of the corresponding LC3B-II (autophagosomes) or mature CTSD (lysosomes) bands. CTSD: Cathepsin D, intermediate (40 kDa) and mature (25 kDa) forms. **d** Quantification of mitochondrial DNA (mtDNA) in the hippocampus from WT and the indicated transgenic mice analyzed by range of age. mtDNA copy numbers were normalized to the copy number of *Bax* and *Gsk3β* genes as a mean of total diploid genome (*n* = 5–11 per range of age and genotype). **e** Expression levels of PGC-1α in total brain extracts. **f** *Tfam* mRNA expression in the hippocampus from WT and the indicated transgenic mice. Transcripts copies were expressed as relative levels referred to the expression in WT mice (*n* = 6). **g** Western blot analysis of TFAM in total brain extracts. **e** and **g** Densitometric values of the bands representing the specific protein immunoreactivity were normalized to the values of the corresponding GAPDH bands (*n* = 6). One-way ANOVA. *P < 0.05; **P < 0.01 (data are mean ± SD). See Supplementary Figure 17 for uncropped blots

Outcomes were slightly different when we analyzed the mitophagy markers in isolated mitochondria from brains of WT and mutant mice. Western blot analysis showed full-length PINK1 only in mitochondria from APP-PSEN1 mice overexpressing SREBF2 (Fig. 3a), whereas levels of the 55 kDa processed form were similar in all the homogenates, regardless of mice genotype (Fig. 3a). Intriguingly, unlike previous observations in cell cultures, mitochondrial accumulation of PINK1 in APP-PSEN1-SREBF2 brains was accompanied by the presence of parkin (Fig. 3a). In contrast, in WT mice and the rest of the mutant mice, parkin was exclusively detected in total brain extracts (Fig. 3a). We examined the levels of lipidated LC3B in isolated mitochondria from WT and mutant mice at increasing ages (Fig. 3b). Unexpectedly, and despite the engagement of the PINK1-parkin pathway, mitochondria from APP-PSEN1-SREBF2 mice did not show recruitment of the autophagosomal marker LC3B at any of the ages analyzed. LC3B-II in mitochondria was only detectable after overexposing the immunoblots, revealing a similar age-dependent increase in both WT and APP-PS1-SREBP2 mice (Supplementary Figure 9). Recruitment of the ULK1 protein kinase complex, a key requirement for mitophagy initiation [42], was also blunted in brains from triple transgenic mice, without any detectable presence of the Ser/Thr kinase ULK1 in the mitochondrial fraction (Fig. 3a). In line with these findings, immunoblots of autophagosomes isolated from brain of APP-PSEN1 mice showed comparable levels of the mitochondrial marker CYC regardless of SREBF2 overexpression (Fig. 3c). Moreover, no differences of CYC levels were observed in the endo-lysosomal fractions between both genotypes (Fig. 3c). Altogether, these findings suggest that the formation of mitophagosomes is disrupted in APP-PSEN1-SREBF2 mice despite PINK1-parkin recruitment. Remarkably, results were different when mitophagy was acutely elicited by an in vivo administration of rapamycin. We observed an increase of CYC in autophagosomes isolated from the brain of rapamycin-treated SREBF2 mice compared to WT mice (Fig. 3c), which was accompanied by low levels of the mitochondrial marker in the endo-lysosomal fraction (Fig. 3c), consistent with the loss of lysosomes fusogenic capacity due to cholesterol rise.

### Impaired mitophagy in APP-PSEN1-SREBF2 brains results in increased mitochondrial content

To further explore whether the cholesterol-mediated impairment of mitophagy affects mitochondrial content in APP-PSEN1-SREBF2 brains, we quantified the mitochondrial DNA (mtDNA) copy number in different brain regions (prefrontal cortex, hippocampus, and cerebellum) by dPCR (Fig. 3d and Supplementary Figure 10). Despite its limitations, dPCR has been shown to correlate with the mitochondrial mass [43]. Results, expressed as mtDNA copies per diploid genome, revealed an age-dependent increase of mtDNA in the hippocampus of APP-PSEN mice that overexpress SREBF2 (Fig. 3d and Supplementary Figure 10). A similar rise was observed in the prefrontal cortex while the mtDNA remained unaltered in the cerebellum, a less affected region in AD (Supplementary Figure 10). In contrast, in the hippocampus of WT and the other transgenic mice, the mtDNA content did not change (APP/PSEN1 mice) or even decreased (WT and SREBF2 mice) at late ages (Fig. 3d).

The increase of mtDNA copies could also reflect in part stimulated mitochondrial biogenesis, triggered by injured mitochondria as a part of the mitochondrial quality control program [44]. To check this possibility, we first determined the expression levels of the co-transcriptional regulation factor PGC-1α /PPARG coactivator 1 alpha (PPARGC1A), a master modulator of mitochondrial biogenesis [45] that through activating the nuclear respiratory factor 1 and 2 (NRF1 and 2) governs the expression of multiple mitochondrial-related proteins, including the mitochondrial transcription factor A (TFAM), ultimately responsible of driving transcription and replication of mtDNA. As shown, immunoblots of brain extract from 7-month-old APP-PSEN1-SREBF2 mice showed a significant increase of PGC-1α levels compared to WT mice (Fig. 3e). Conversely, the transcriptional regulatory factor was found reduced in brain extracts of 10-month-old APP-PSEN1 mice with and without SREBF2 overexpression (Fig. 3e). In agreement with these findings, mRNA levels of *Tfam* significantly increased only in brains of 7-month-old APP-PSEN1-SREBF2 mice (Fig. 3f), which was associated with an increase of the TFAM protein levels (Fig. 3g). No significant changes in TFAM expression levels were observed in WT mice and the rest of mutant mice, at any of the ages analyzed (Fig. 3f and g). Thus, these results indicate that while the increase of the mtDNA content in the brain of APP-PSEN1-SREBF2 mice up to 7 months of age is in part due to biogenesis induction, the mtDNA rise at late ages can only be accounted for mitophagy defects.

### Progressive activation of the PINK1-parkin signaling pathway in brain mitochondria from APP-PSEN1-SREBF2 mice during aging

**Fig. 4:**
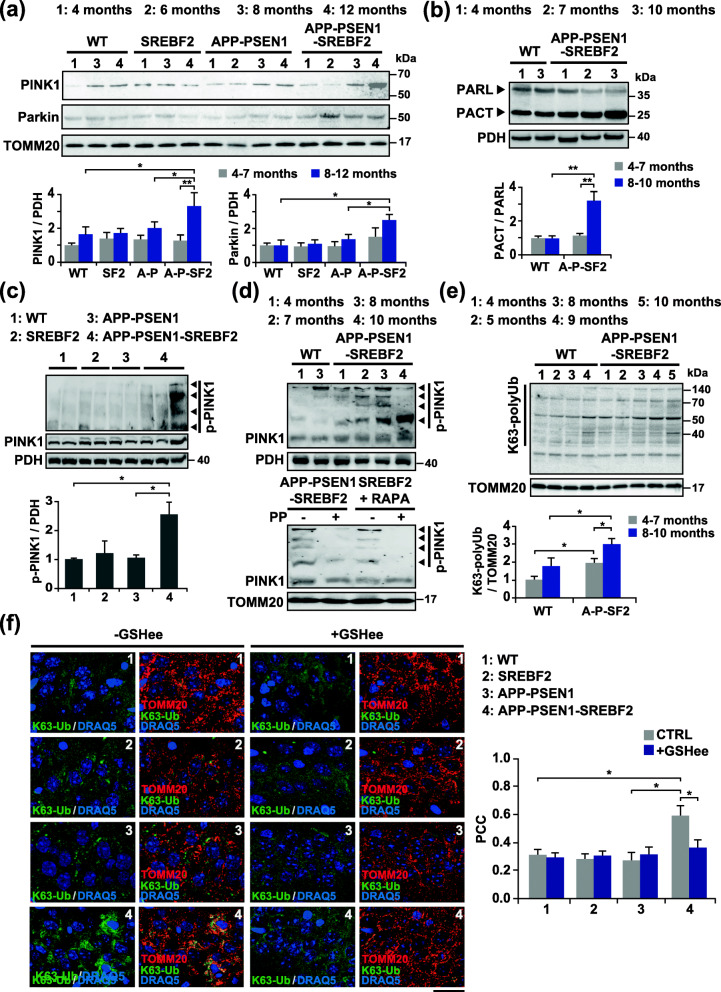
Age-dependent activation of the PINK1-parkin pathway in mitochondria from brains of APP-PSEN1-SREBF2 mice. Mitochondria-rich fractions were isolated from brain extracts of WT and the indicated transgenic mice at increasing ages. **a** and **b** Representative immunoblots showing the expression levels of PINK1, parkin and PARL in the mitochondria-rich fraction. PACT: C-terminal PARL fragment. **c** and **d** Phos-tag™ SDS-PAGE analysis of phospho-PINK (p-PINK1) in the mitochondria-rich fraction from WT and the indicated transgenic mice. As a control, samples were treated with 50 U of alkaline phosphatase (PP) for 1 h at 37 °C to inhibit phosphorylation. SREBF2 mice were treated with the autophagy inducer rapamycin (RAPA, 5 mg/kg, i.p.) for 24 h. PDH and TOMM20 were used to confirm equal loading. **e** Representative K63 polyubiquitin (K63-polyUb) western blot. K63-linkage specific polyubiquitin antibodies were used to detect K63 ubiquitination of proteins in the mitochondrial fractions. During mitochondria isolation, deubiquitination of the proteins was prevented by including 10 mM N-ethylmaleimide (NEM) in the isolation buffer. **a**, **b**, **c**, and **e** Densitometry of the bands representing specific protein immunoreactivity was assessed from samples grouped in the indicated range of age and values were normalized to the corresponding PDH or TOMM20 values. C-terminal PARL (PACT) fragment values were normalized to unprocessed PARL values (*n* = 3–4 per range of age and genotype). SF2: SREBF2 mice, A-P: APP-PSEN1 mice, A-P-SF2: APP-PSEN1-SREBF2 mice. See Supplementary Figure 18 for uncropped blots. **f** Hippocampal slices from 10-month-old WT and the indicated transgenic mice. Shown are confocal photomicrograph of K63-polyUb (green) and TOMM20 (red) immunoreactivity. To recover the mitochondrial GSH content, mice were treated with 1.25 mmol/kg of GSH ethyl ester (GSHee) every 12 h for 2 weeks. Nuclei were counterstained by DRAQ5 (blue). Scale bars: 25 μm. The Pearson’s correlation coefficient (PCC) was calculated from 3 random fields per condition. One-way ANOVA. *P < 0.05; **P < 0.01 (data are mean ± SD)

Having established that mitophagy flux is impaired in APP-PSEN1-SREBF2 mice and results in increased mitochondrial content, we next sought to go deeper insight into the nature of these cholesterol-mediated alterations during aging, focusing our analyses mainly on the initial steps of the PINK1-parkin cascade of events. We examined the presence of PINK1 and parkin in isolated mitochondria from brains of WT and mutant mice at increasing ages (Fig. 4a). As shown, only mitochondria from APP-PSEN1-SREBF2 mice displayed a significant age-dependent accumulation of PINK1 that was accompanied by enhanced parkin recruitment (Fig. 4a).

In healthy mitochondria, PINK1 is targeted to the inner mitochondrial membrane and cleaved by the rhomboid intra-membrane protease PARL (presenilin associated, rhomboid-like). In some cases, when PARL activity becomes rate limiting, the unprocessed PINK1 can reach again the OMM and initiate mitophagy, without loss of mitochondrial membrane potential [46]. Interestingly, recent studies have shown that mitochondrial bioenergetic stress promotes an autocatalytic cleavage of PARL, producing a C-terminal PARL (PACT) fragment less efficient in processing PINK1 [47]. We analyzed the presence of PARL and the resulting processed PACT fragments in mitochondrial extracts by western blot (Fig. 4b). The immunoblots revealed an age-dependent increase of the PACT/PARL ratio in mitochondria from brains of APP-PSEN1-SREBF2 mice, while mitochondria from WT mice displayed a similar PACT/PARL ratio, regardless of aging. It is therefore likely that the increment of this proteolytically deficient fragment of PARL in mitochondria of triple transgenic mice may favor the observed rise of PINK1 levels, although, further studies would be need to prove a mechanistic link.

Next, as a measure of PINK1 functionality, we examined its phosphorylation state in mitochondrial extracts by phosphate affinity SDS-polyacrylamide gel electrophoresis (Phos-tag SDS-PAGE), an assay based on the ability of the Phos-tag reagent to bind phosphorylated proteins and retard their electrophoretic mobility [48]. Phos-tag immunoblots showed an increased presence of slower migration bands in brain mitochondrial extracts from 7-month-old APP-PSEN1-SREBF2 mice compared to APP-PSEN1 samples (Fig. 4c) and as expected, the bands disappeared after phosphatase treatment (Fig. 4d). The shifted bands were more evident in older APP-PSEN1-SREBF2 mice, indicating a progressive accumulation of hyperphosphorylated PINK1(Fig. 4d). To further confirm that the PINK1-parkin signaling axis is active, we analyzed the levels of polyubiquitinated chains, in particular those connected via lysine residues at position 63 (K63), which act as scaffolds for autophagy receptors [49, 50]. Immunoblots displayed an increased age-dependent accumulation of high molecular weight bands in brain mitochondrial extracts from APP-PSEN1-SREBF2 mice in comparison with samples from WT and APP-PSEN1 mice (Fig. 4e and Supplementary Figure 11), which disappeared in the absence of the deubiquitinases inhibitor N-ethylmaleimide (NEM) (Supplementary Figure 11). Immunostaining analyses of hippocampal slices from APP-PSEN1-SREBF2 mice corroborated the increased levels of K63-ubiquitin in mitochondria, showing high co-localization with the translocase of outer mitochondrial membrane 20 (TOMM20) (Fig. 4f). Remarkably, the ubiquitin recruitment to mitochondria was completely abolished following an in vivo treatment with GSHee for 2 weeks (Fig. 4f). Thus, these findings suggest that high brain cholesterol levels in APP-PSEN1-SREBF2 mice stimulate the PINK1-parkin-mediated ubiquitin signaling by modulating oxidative stress.

### Reduced mitochondrial translocation of OPTN in elderly APP-PSEN1-SREBF2 mice due to accumulation in aggresome-like structures

**Fig. 5:**
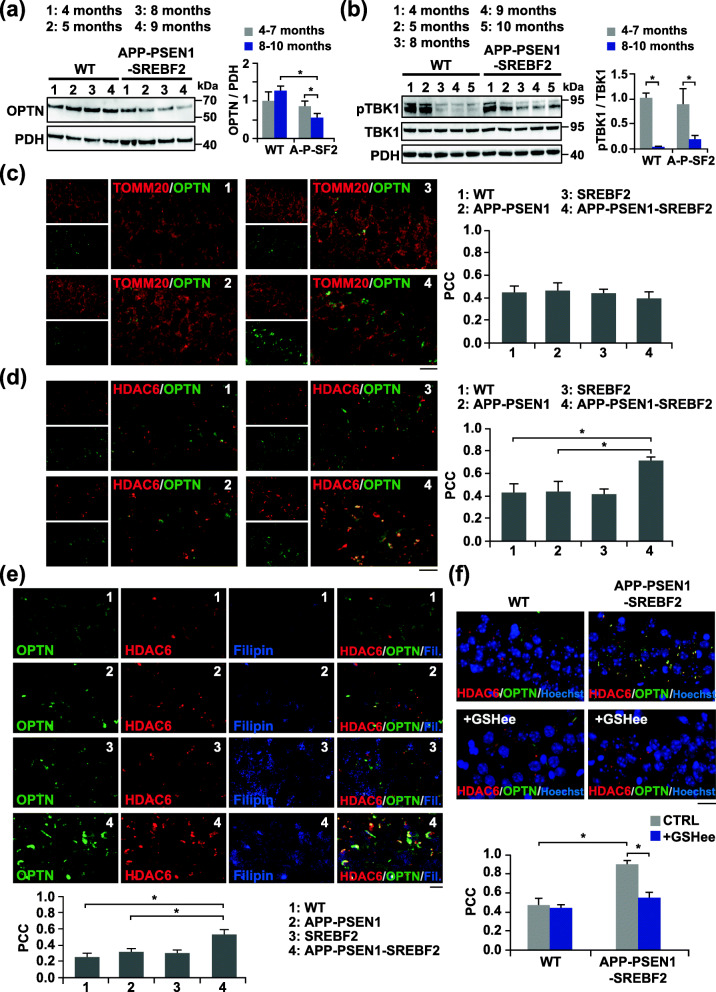
Reduced recruitment of OPTN to mitochondria that correlates with oxidative-induced OPTN-positive aggresomes in old APP-PSEN1-SREF2 mice. **a** and **b** Representative immunoblots of OPTN levels (**a**) and the protein levels of TBK1 and its phosphorylated form (pTBK1) (**b**) in the mitochondrial fraction isolated from brain extracts of WT and APP-PSEN1-SREBF2 mice at increasing ages. Densitometry of the bands representing specific protein immunoreactivity was assessed from samples grouped in the indicated range of age and values were normalized to the corresponding PDH values. pTBK1 values were normalized to TBK1 values. (*n* = 4 per range of age and genotype). A-P-SF2: APP-PSEN1-SREBF2 mice. See Supplementary Figure 19 for uncropped blots. **c-f** Hippocampal slices from 10-month-old WT and the indicated transgenic mice. Shown are photomicrograph of OPTN (green) and TOMM20 (red) immunoreactivity (**c**) or OPTN (green) and HDAC6 (red) immunoreactivity (**d**-**f**) with filipin staining (**e**). To recover the mitochondrial GSH content, mice were treated with 1.25 mmol/kg of GSH ethyl ester (GSHee) every 12 h for 2 weeks. Nuclei were counterstained by bisBenzimide Hoechst 33258 (blue). Scale bars: 25 μm. The Pearson’s correlation coefficient (PCC) was calculated from 3 independent experiments (at least 4 random fields were analyzed per condition). One-way ANOVA. *P < 0.05; **P < 0.01 (data are mean ± SD)

The next step was to evaluate the presence of autophagy receptors in mitochondria. As previously shown, SQSTM1 accumulated in APP-PSEN1-SREBF2 neurons (Supplementary Figure 8b), and also in brain mitochondria of old triple transgenic mice (Supplementary Figure 12a). Nonetheless, according to previous studies, the role of SQSTM1 is limited to mitochondrial clustering [51] while OPTN and NDP52 would be the primary PINK1-parkin-dependent mitophagy receptors [50, 52]. Moreover, despite functional redundancy, OPTN and NDP52 are differentially distributed among tissues, being OPTN highly expressed in the brain [52]. These observations prompted us to study the impact of high cholesterol particularly on the recruitment of OPTN to mitochondria. We observed that levels of OPTN increased in total brain homogenates from APP-PSEN1-SREBF2 mice (Supplementary Figure 12b). Conversely, the presence of the autophagy receptor was markedly lower in the mitochondrial fraction of the triple transgenic mice, in comparison with samples from WT and the other mutant mice (Supplementary Figure 12b). OPTN content progressively decreased with age in brain mitochondria of APP-PSEN1 mice overexpressing SREBF2 (Fig. 5a). In contrast, Aβ-treated SH-SY5Y cells acutely enriched with cholesterol displayed enhanced mitochondrial levels of OPTN, and the consequent recruitment of LC3B-II (Supplementary Figure 12c), consistent with the observed formation of mitophagosomes (Fig. 1c). Thus, these findings indicate that chronic cholesterol accumulation in the brain of APP-PSEN1-SREBF2 mice results in an age-dependent impairment of OPTN translocation to mitochondria.

Given that phosphorylation of OPTN by TANK-binding kinase-1 (TBK1) has been described to promote its binding to ubiquitin, thereby favoring its retention to damaged mitochondria [53], we investigated whether the decreased mitochondrial levels of OPTN in APP-PSEN1-SREBF2 mice correlate with a limited phosphorylation-mediated activation of TBK1. Western blot analysis showed a significant decrease of phosphorylated TBK1 in brain extracts of old APP-PSEN1-SREBF2 mice; nonetheless, the reduction was even more pronounced in brains from WT mice (Fig. 5b), indicating that the lower presence of OPTN in mitochondria of triple transgenic mice cannot be explained simply by age-dependent changes in TBK1 activity.

Expression and distribution of OPTN in mouse brains were also monitored by fluorescence microscopy (Fig. 5c). Immunostainings with anti-OPTN yielded a higher intensity in the hippocampus of 10-month-old APP-PSEN1-SREBF2 mice compared to WT and APP-PSEN1 brains (Fig. 5c). Nonetheless, the autophagy receptor did not co-localize with the mitochondrial marker TOMM20 but accumulated in cytosolic deposits (Fig. 5c). Cellular inclusions of mutant OPTN are a hallmark of amyotrophic lateral sclerosis (ALS) and primary open-angle glaucoma (POAG) [54]. Likewise, intraneuronal aggregates of OPTN have been observed in other neurodegenerative diseases, including AD [55]. Under some pathological conditions and stimuli like oxidative stress, OPTN can form aggregates made of covalently-bonded oligomers [56]. Moreover, if the ubiquitin–proteasome system fails these aggregates are sequestered into large inclusion body-like structures called aggresomes [57], which can eventually be eliminated via autophagy [58]. To confirm that the cytosolic buildups of OPTN in hippocampal neurons of APP-PSEN1-SREBF2 mice were part of aggresome-like structures, we analyzed their interaction with the histone deacetylase 6 (HDAC6), a crucial player in the recruitment of polyubiquitinated proteins and the subsequent aggresome formation [59]. Consistent with a stimulated aggresome synthesis, photomicrographs of the hippocampus from 10-month-old triple transgenic mice showed an increased presence of HDAC6 that co-localized with OPTN (Fig. 5d). Furthermore, filipin staining of APP-PSEN1-SREBF2 brain slices showed a marked presence of cholesterol in the OPTN-containing aggresomes; in contrast, intracellular cholesterol staining was almost negligible in WT and APP-PSEN1 brain samples (Fig. 5e). In all the cases, cell membranes were not labeled by filipin since samples were permeabilized before OPTN and HDAC6 immunostaining. Interestingly, the formation of OPTN-containing aggresomes was significantly reduced when mice were subjected to an in vivo treatment with GSHee (Fig. 5f), indicating that sustained cholesterol-mediated depletion of mitochondrial GSH and the subsequent oxidative stress in aged APP-PSEN1-SREBF2 mice may play a key role in regulating OPTN expression and its recruitment to aggresomes.

### Progressive presence of OPTN-containing aggresomes in the hippocampus of AD patients correlates with increased mitochondrial cholesterol levels at late stages

**Fig. 6:**
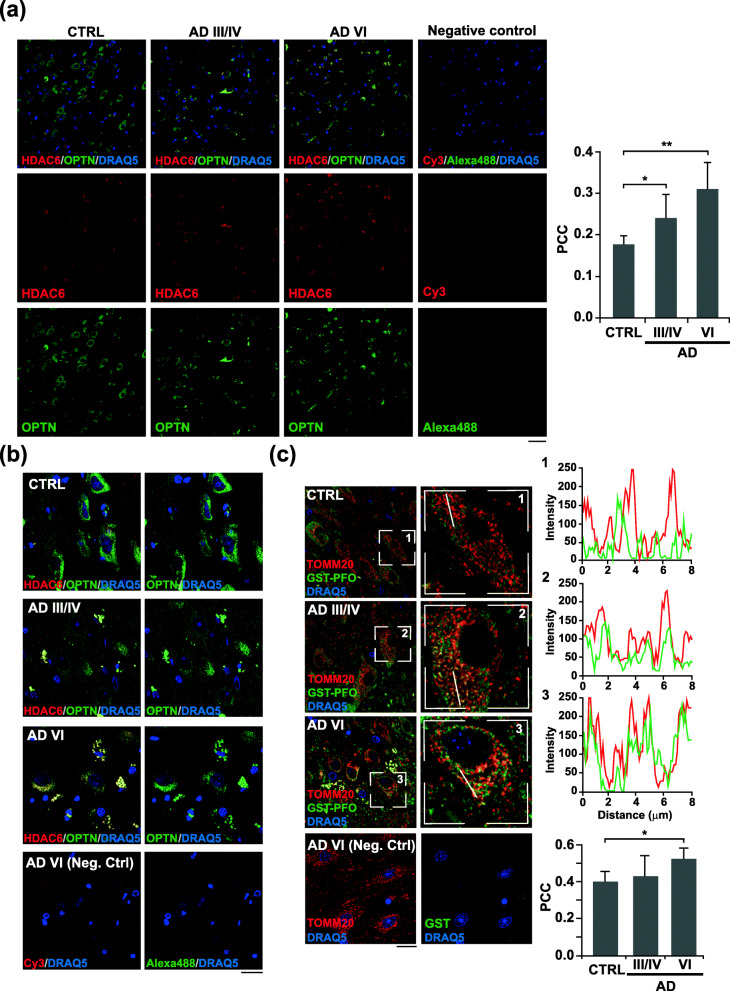
Pyramidal neurons in the hippocampus from AD patients display OPTN in HDA6C-positive aggregates associated with high mitochondrial cholesterol levels. **a** and **b** Hippocampal slices from control (CTRL) and AD patients classified into three groups following the “ABC” score: CTRL, intermediate AD (AD III-IV) and high AD (AD VI). Shown are representative confocal photomicrograph of double immunofluorescence for OPTN (green) and HDAC6 (red) at 20X magnification (**a**) and 63X magnification (**b**). A negative control slide incubated only with secondary antibodies was included. **c** Shown are representative confocal photomicrograph of double immunofluorescence for GST-PFO (green) and TOMM20 (red). Sections were incubated with 20 μg/ml GST-PFO for 3 h prior immunolabeling. Insets show a 3-fold magnification of the indicated regions. A negative control without GST-PFO incubation was included. Graphs depict the intensity profiles of GST-PFO and TOMM20 fluorescence in the cross-section. Nuclei were counterstained by DRAQ5 (blue). Scale bar: 50 μm (**a**) and 25 μm (**b** and **c**). In all the cases, the Pearson’s correlation coefficient (PCC) was calculated from 5 individuals per group (at least 6 random fields were analyzed per sample). *P < 0.05; **P < 0.01 (data are mean ± SD)

Recently, Fang et al. [12] have provided compelling evidence of mitophagy impairment in the hippocampus of AD patients. In line with our findings, the study showed low levels of mitophagy initiation proteins, such as phosphorylated TBK1 and ULK1, in all the human AD samples analyzed. They also reported unchanged OPTN levels in total brain extracts; however, the intracellular distribution of OPTN was not specifically evaluated. We addressed this question by analyzing the presence of OPTN-containing aggresomes in post-mortem hippocampal tissues from age-matched AD and control individuals (patients’ information in Table 1). The immunohistochemical analysis showed HDAC6-positive structures in the hippocampus of individuals with AD, which increased progressively along AD stages and displayed a higher degree of co-localization with OPTN in comparison with tissue from control subjects (Fig. 6a and Supplementary Figure 13). At higher magnification, confocal micrographs from control tissues showed an OPTN immune-positivity evenly distributed throughout the neuronal soma (Fig. 6b). In contrast, a perinuclear accumulation of the autophagy receptor was noticed in pyramidal neurons of AD tissues, which progressed towards more condensed aggresome-like structures in the late AD VI stages (Fig. 6b). Thus, these results corroborated the observations in APP-PSEN1-SREBF2 mice, which led us to question whether OPTN aggregates in human AD tissues were also linked to changes in cholesterol levels. To avoid the reported drawbacks of filipin use in immunohistochemistry of human brain tissues (photobleaching issues and high autofluorescence at the filipin excitation wavelength), we analyzed the cholesterol distribution in hippocampal slices using recombinant perfringolysin O, a cholesterol-binding bacterial toxin fused with glutathione S-transferase (GST-FPO). We first performed a protein-lipid overlay assay to confirm that the recombinant protein recognizes cholesterol. As shown, GST-PFO (1 μg/ml) binds cholesterol in a dose-dependent manner, starting from 200 pmol of the sterol (Supplementary Figure 14a). The selectivity of the probe for cholesterol was also demonstrated in SH-SY5Y cells, showing an enhanced GST-PFO fluorescence intensity after cholesterol enrichment that mimicked the cellular pattern of filipin staining (Supplementary Figure 14b). In the hippocampus of human control brains, GST-PFO immunofluorescence staining mainly visualized the boundaries of the pyramidal neurons, consistent with the high cholesterol content of the plasma membrane (Supplementary Figure 15). Cholesterol labeling at the plasma membrane significantly decreased in hippocampal neurons from AD brains, turning into more intracellular staining, with GST-PFO-immunopositive aggregates at late AD VI stages (Supplementary Figure 15). Furthermore, quantification of confocal microscopy images showed significant co-localization of GST-PFO with TOMM20 in hippocampal neurons of AD-affected brains at late stages (Fig. 6c), suggesting that cholesterol accumulates in mitochondria throughout the course of the disease.

Overall, our data illustrate systematically the impact of high cholesterol levels in Aβ-induced mitophagy. Cholesterol exerts a dual effect; it promotes PINK1-mediated mitophagy induction by downregulating the mitochondrial antioxidant defense but impairs the lysosomal clearance of mitophagosomes. Additionally, during the disease progression, a chronic high cholesterol loading stimulates the oxidative-mediated formation of OPTN aggregates, thereby inhibiting its translocation to mitochondria and the mitophagy completion, despite enhanced PINK1/parkin activation.

## Discussion

Our data provide evidence that increased cholesterol burden in the brain interferes with the ubiquitin-mediated mitophagy process, contributing to the impaired mitochondrial clearance reported in AD. We had previously established a mechanistic link between high brain cholesterol and impaired autophagy in AD, showing that cholesterol regulates autophagy through a dual mechanism that elicits autophagosome formation by stimulating the oxidative-mediated lipidation of LC3B but impairs the autophagosome-lysosome fusion by affecting the recycling of key proteins in the fusion process [18]. The resulting blockage of the autophagy flux affected intracellular MAPT/tau and Aβ clearance and favored the release of Aβ via an unconventional autophagy-mediated secretory pathway [18]. In the present work, we unveil the regulatory role of cholesterol in mitochondria clearance. Cholesterol-mediated downregulation of the antioxidant mitochondrial defense triggers the activation of the PINK1-parkin signaling pathway that culminates in an incomplete mitochondrial degradation due to defective lysosomal resolution. Moreover, in aged mice, chronic cholesterol accumulation results in an age-dependent impairment of OPTN translocation to mitochondria, inhibiting mitophagosomes formation despite enhanced PINK1/parkin activation. The role of lipids in the control of the PINK1-parkin pathway was previously suggested in studies where mice fed with a high-fat and -cholesterol diet showed a marked increase in the hepatic levels of parkin [60]. Additionally, using a genome-wide RNAi screening, different lipogenesis-related genes have been identified as regulators of the PINK1-parkin signaling axis, highlighting the role of SREBF1 in promoting mitochondrial PINK1 stabilization after mitophagy induction [61]. In the same line, our findings show that neuronal cholesterol rise favors the accumulation of PINK1 in mitochondria in response to Aβ, an event followed by parkin recruitment in brains of APP-PSEN1 mice overexpressing SREBF2.

The involvement of the ubiquitin-mediated pathway of mitophagy is well established in neuronal cultures, with studies showing its activation under basal conditions, even without overexpressing PINK1 or parkin [62], and in response to mitochondrial stress [63]. In contrast, its role in vivo has been a matter of debate, particularly in tissues with high metabolic demand like the brain, where basal mitophagy appears to proceed independently of PINK1 [64]. Moreover, neither parkin- nor PINK1-KO mice exhibit severe neurodegenerative symptoms, which would further question the physiological significance of the pathway [65, 66]. However, recent works have shed some light on this issue by showing that PINK1-parkin-mediated mitophagy becomes relevant in response to intense mitochondrial stress conditions [64, 67]. Based on these findings, and our previous studies showing that cholesterol in brains from APP-PSEN1-SREBF2 mice exacerbates Aβ-induced oxidative damage [26, 27], it is, therefore, reasonable to assume that long-term cholesterol loading may lead to a scenario of chronic oxidative stress able to surpass the activating threshold of PINK1-parkin pathway. A threshold that would not be reached in APP-PSEN1 mice. The involvement of cholesterol-enhanced mitochondrial ROS in the activation of the ubiquitin-dependent pathway is supported by results from cholesterol-enriched cultures exposed to Aβ and in in vivo studies using GSHee. In neuronal cultures, the presence of mitochondrial PINK1 and mitophagosomes formation is completely abolished by pretreatment with GSHee, a membrane-permeable derivative of GSH that we have previously demonstrated to recover cholesterol-induced mitochondrial GSH depletion and inhibit Aβ-stimulated ROS [18, 26]. Furthermore, we also show that in vivo treatment of GSHee for 2 weeks prevents the mitochondrial recruitment of K63-ubiquitin associated with the high presence of phosphorylated PINK1 in the hippocampus of old APP-PSEN1-SREBF2 mice. These data complement previous studies that illustrate how modulation of the mitochondrial antioxidant protein superoxide dismutase-2 has a direct impact on mitophagy progression, and point to mitochondrial ROS production as an upstream inducer of the process [63, 68, 69].

In primary neuronal cultures that overexpress SREBF2, Aβ induces mitophagy independently of parkin. Lazarou et al. have demonstrated [52] that PINK1 can recruit the autophagy receptors NDP52 and OPTN to mitochondria without parkin activation, suggesting that the E3 ubiquitin ligase is dispensable for mitophagy, and only acts to amplify the PINK1-generated phospho-ubiquitin signal. Parkin-independent mitophagy has also been reported in neurons exposed to ionophores such as CCCP [38]. These results appear to be at odds with the mitochondrial recruitment of parkin that we observed after mitochondria depolarization by valinomycin. This apparent discrepancy may be likely related to the inducer used and the specific time course of mitophagy in neurons, which is longer compared with non-neuronal cells [70, 71]. Besides, we have shown that Aβ induces mitochondrial accumulation of PINK1 without loss of membrane potential, in agreement with recent studies indicating that depolarization is not a sufficient condition for eliciting mitophagy in neurons [72]. The mechanism is not completely clear. Nevertheless, studies from Jin et al. [73] have shown that mitochondrial accumulation of unfolded proteins can induce PINK1 stabilization in polarized mitochondria. In addition, both APP and Aβ have been described in mitochondria, clogging the TOM complex and thereby affecting the mitochondrial import competence [74, 75], which in turn can trigger the accumulation of active PINK1 in the OMM [76]. Interestingly, high-fat diet has been described to promote APP miss-localization to mitochondria in white adipose tissue [75]. Therefore, it is conceivable to think that increased cholesterol levels may contribute in part to PINK1 stabilization in mitochondria by favoring APP/Aβ deposition, although experimental confirmation would be needed to support such a mechanism in neurons. On the other hand, the loss of PARL, the protease responsible for PINK1 cleavage at the IMM, has also been reported to phenocopy the mitophagy induced by uncoupling agents [46]. PARL undergoes an N-terminal autocatalytic cleavage (β cleavage), resulting in a less active form (PACT fragment). This proteolytic cleavage is required for efficient mitophagy and acts as a sensor of bioenergetic changes [47]. Accordingly, altered PARL function due to mutations on the β cleavage site has been reported to contribute to mitochondrial dysfunction in PD [77]. Our immunoblots show enhanced processing of PARL in brain mitochondria from aged APP-PSEN1-SREBF2 mice that correlates with PINK1 accumulation, suggesting that cholesterol loading may regulate mitochondrial PINK1 stability by inducing the β cleavage of the protease, most likely in response to mitochondrial stress [47].

Using the ratiometric fluorescent protein mt-mKeima to monitor mitophagy flux in live cells, we have observed that the delivery of mitochondria to lysosomes is disrupted in cholesterol-enriched SH-SY5Y cells, in agreement with our previous studies showing that the increase of cholesterol in lysosomes affects its fusogenic capacity [18]. Similarly, in Niemann Pick type C1 (NPC1)-deficient neurons, which display an abnormal distribution of cholesterol due to sequestration in the lysosomal compartment, autophagy progression is hampered and correlates with a defective mitochondrial clearance [78]. The link between altered lipid homeostasis and impaired mitophagy resolution is also suggested by a study that monitored mitophagy in the liver of transgenic mice expressing mt-Keima and reported a reduced mitophagy flux when mice were fed a high-fat diet from 18 to 20 weeks [79]. Interestingly, our previous data showed that administration of the hydroxypropyl form of β-cyclodextrin (HP-β-CD), a cyclic polysaccharide compound that modulates the cellular cholesterol content, recovers defective autophagosome-lysosome fusion and restores the impaired Aβ and MAPT/tau clearance in APP-PSEN1-SREBF2 mice [18]. An enhancement of lysosomal dynamics and autophagy has also been reported in HP-β-CD-treated NPC1 mice [80]. Therefore, treatment with HP-β-CD may also benefit mitophagy.

Unlike cell cultures, the long-term alteration of cholesterol homeostasis in the brain of APP-PSEN1-SREBF2 mice stimulates PINK1 and parkin activation but fails to recruit the core autophagy proteins ULK1 and LC3B to form the mitophagosome. The disruption of the mitophagy flux correlates with increased mtDNA copy number, assessed as an indicator of mitochondrial content. In apparent contradiction with these findings, low DNA copy numbers have been reported in brains from AD-affected individuals [81, 82]. However, it is worth noting that when the brain mtDNA content is analyzed in AD patients with or without diabetes, the mtDNA displays upregulated only in those suffering from diabetes [83], a metabolic disorder frequently underlying the neurodegenerative process and that has been linked to enhanced cholesterol synthesis [84].

We show an age-dependent reduction of OPTN in mitochondria from APP-PSEN1-SREBF2 brains, associated with the appearance of OPTN in cytosolic aggregates that co-localize with the aggresome-related protein HDAC6. Furthermore, recovery of the mitochondrial pool of GSH, following 2 weeks of in vivo GSHee treatment, prevents the presence of these aggresome-like structures, which corroborates previous data showing enhanced oxidative stress as the trigger of OPTN oligomerization [56] and the maintenance of GSH homeostasis as a key event to prevent abnormal accumulation of protein aggregates [85]. HDAC6 in cooperation with autophagy receptors mediates the clearance of these aggregates via autophagy [86]. Strikingly, filipin staining reveals an enhanced cholesterol loading in some of the OPTN-containing aggresomes, which may be explained by the presence of autophagosome membranes and an incomplete lysosomal degradation. Cholesterol in APP-PSEN1-SREBF2 brains not only accumulates in mitochondria, but also in autophagic vesicles and endo-lysosomal membranes, reducing its fusogenic capacity [18]. Therefore, cholesterol by blocking the autophagy resolution may also contribute to increase the presence of HDAC6-positive aggresome in AD brains. Accordingly, HDAC6 expression has been reported to be upregulated by 90% in AD hippocampus, co-localizing with MAPT/tau in perinuclear aggresomes [87], and despite its potential protective role in protein aggregates disposal, loss of HDAC6 has been shown to improve AD symptoms in transgenic mouse models [87].

To our knowledge, these findings show for the first time a progressive accumulation of OPTN in HDAC6-positive aggregates in post-mortem AD brains that correlates with an increase of mitochondrial cholesterol levels with disease progression, thus, providing a mechanistic explanation of the reported mitophagy disruption in AD patient brains [12]. Apart from its role in mitophagy, OPTN has been shown to inhibit the receptor-interacting kinase 1 (RIPK1)–dependent signaling [88], therefore, inactivation of OPTN in aggregates may also contribute to neuronal death by unleashing the necroptosis signaling pathway. High mitochondrial cholesterol content has also been reported within astrocytes of brains from AD-affected individuals, associated with an enhanced expression of the mitochondrial cholesterol carrier STARD1 [89], in line with former studies that described stimulated steroidogenesis in AD brains [90]. The cause that promotes this abnormal transport of cholesterol to mitochondria is not fully known, however, data from experimental AD models suggest the involvement of Aβ-induced ER stress [91].

## Conclusions

Our studies reveal a novel mechanism connecting cholesterol-induced mitochondrial oxidative stress with reduced mitochondrial clearance and AD progression. Cholesterol exerts a dual effect on PINK1-parkin-mediated mitophagy, by impairing lysosomal clearance of mitophagosomes and promoting a progressive oxidative-induced accumulation of OPTN aggregates that prevents its mitochondrial recruitment despite PINK1/parkin activation. The data highlight the relevance of using specific brain cholesterol-lowering strategies to recover lysosomal function and restrain excessive mitochondrial ROS, which in combination with autophagy inducers may be of significance in the treatment of AD.

## Supplementary Information

**Additional file 1: Figure S1.** Upregulated mRNA expression levels of cholesterol-related genes in hippocampus from mice that overexpress SREBF2. **Figure S2.** Cross-contamination analysis of autophagosomal (F1) and lysosomal (F3) fractions isolated by density gradient centrifugation. **Figure S3.** Cholesterol enrichment in SH-SY5Y cells prevents mitochondrial lysosomal clearance after Aβ induction of mitophagy. **Figure S4.** Impaired mitophagy flux in cholesterol-enriched cells incubated with CCCP. **Figure S5.** Cell Viability of WT and SREBF2 neurons was not affected by the treatment with Aβ, rapamycin or GSH ethyl ester (GSHee). **Figure S6.** Defective mitophagy in SREBF2 neurons exposed to rapamycin. **Figure S7.** Mitochondrial membrane potential remains unchanged in cultured primary neurons after Aβ exposure. **Figure S8.** SREBF2 overexpression in APP-PSEN1 neurons results in an accumulation of mitophagosomes but prevents mitophagy completion. **Figure S9.** Immunoblot analysis of LC3B levels in mitochondria isolated from brains of WT and APP-PSEN1-SREBF2 mice from 4, 7, and 10 months of age. **Figure S10.** Quantification of mitochondrial DNA (mtDNA) in the prefrontal cortex (PFC), hippocampus (HP), and cerebellum (CB) from APP-PSEN1-SREBF2 mice analyzed by range of age. **Figure S11.** Mitochondria from the brains of 7-month-old APP-PSEN1-SREBF2 mice display enriched content of K63 polyubiquitinated proteins. **Figure S12.** Differential recruitment of autophagy receptors in brains of APP-PSEN1-SREBF2 mice and in cholesterol-enriched SH-SY5Y cells exposed to Aβ. **Figure S13.** Appearance of OPTN-positive aggregates in CA3-CA2 hippocampal layers concomitant to the neuropathological AD progression. **Figure S14.** Selective recognition of cholesterol by GST-PFO. **Figure S15.** Differential cholesterol distribution in hippocampal neurons with the progression of neuropathological AD stages. **Figure S16.** Uncropped scans of western blots included in Fig. 1. **Figure S17.** Uncropped scans of western blots included in Fig. 3. **Figure S18.** Uncropped scans of western blots included in Fig. 4. **Figure S19.** Uncropped scans of western blots included in Fig. 5.

## Data Availability

Data and materials are available from the corresponding author on reasonable request.

## Abbreviations

Aβ: Amyloid beta
ACTB: Beta-actin
AD: Alzheimer’s Disease
APP: Amyloid precursor protein
CAA: Cerebral amyloid angiopathy
CB: Cerebellum
CCCP: Carbonyl cyanide chlorophenylhydrazone
CHO:MCD: Cholesterol:methyl-β-cyclodextrin
CQ: Chloroquine
CTSD: Cathepsin D
CYC: Cytochrome C
dPCR: Digital polymerase chain reaction
GAPDH: Glyceraldehyde-3-phosphate dehydrogenase
GSH: Glutathione
GSHee: Glutathione ethyl ester
GST: Glutathione-S-transferase
HDAC6: Histone deacetylase 6
HP: Hippocampus
IMM: Inner mitochondrial membrane
LAMP2: Lysosome-associated membrane glycoprotein 2
MAP 1 LC3B: Microtubule-associated protein 1 light chain 3B
MAPT: Microtubule-associated protein tau
mtDNA: Mitochondrial DNA
mTOR: Mechanistic target of rapamycin
NEM: N-ethylmaleimide
NFT: Neurofibrillary tangles
NDP52: Nuclear domain 10 protein 52 kDa
NPC1: Niemann-Pick type C1
OMM: Outer membrane of mitochondria
OPTN: Optineurin
OXPHOS: Oxidative phosphorylation
PACT: C-terminal PARL
PARL: Presenilins-associated rhomboid-like protein
PCC: Pearson’s correlation coefficient
PDH: Pyruvate dehydrogenase
PFC: Prefrontal cortex
PFO: Perfringolysin O
PGC-1α: PPAR-gamma coactivator 1-alpha
PINK1: PTEN-induced putative kinase protein 1
PMI: Post-mortem interval
PS: Ponceau S
PSEN1: Presenilin 1
RAPA: Rapamycin
RIPK1: Receptor-interacting protein kinase 1
ROS: Reactive oxygen species
SQSTM1: Sequestosome-1
SREBF2: Sterol regulatory element binding factor 2
STARD1: Steroidogenic acute regulatory (STAR) domain-containing protein 1
TBK1: TANK-binding kinase-1
TFAM: Mitochondrial transcription factor A
TOMM20: Translocase of outer mitochondrial membrane 20
ULK1: Unc-51-like kinase 1
WT: Wild-type

## References

[1] Spinelli JB, Haigis MC (2018). The multifaceted contributions of mitochondria to cellular metabolism. Nat Cell Biol.

[2] Rangaraju V, Calloway N, Ryan TA (2014). Activity-driven local ATP synthesis is required for synaptic function. Cell.

[3] Gusdon AM, Chu CT (2011). To eat or not to eat: neuronal metabolism, mitophagy, and Parkinson's disease. Antioxid Redox Signal.

[4] Kawamata H, Manfredi G (2017). Proteinopathies and OXPHOS dysfunction in neurodegenerative diseases. J Cell Biol.

[5] Grimm A, Friedland K, Eckert A (2016). Mitochondrial dysfunction: the missing link between aging and sporadic Alzheimer's disease. Biogerontology.

[6] Hauptmann S, Scherping I, Drose S, Brandt U, Schulz KL, Jendrach M, Leuner K, Eckert A, Muller WE (2009). Mitochondrial dysfunction: an early event in Alzheimer pathology accumulates with age in AD transgenic mice. Neurobiol Aging.

[7] Vanhauwaert R, Bharat V, Wang X (2019). Surveillance and transportation of mitochondria in neurons. Curr Opin Neurobiol.

[8] Harper JW, Ordureau A, Heo JM (2018). Building and decoding ubiquitin chains for mitophagy. Nat Rev Mol Cell Biol.

[9] Pickles S, Vigie P, Youle RJ (2018). Mitophagy and quality control mechanisms in mitochondrial maintenance. Curr Biol.

[10] Rodolfo C, Campello S, Cecconi F (2018). Mitophagy in neurodegenerative diseases. Neurochem Int.

[11] Valente EM, Abou-Sleiman PM, Caputo V, Muqit MM, Harvey K, Gispert S, Ali Z, Del Turco D, Bentivoglio AR, Healy DG (2004). Hereditary early-onset Parkinson’s disease caused by mutations in PINK1. Science.

[12] Fang EF, Hou Y, Palikaras K, Adriaanse BA, Kerr JS, Yang B, Lautrup S, Hasan-Olive MM, Caponio D, Dan X (2019). Mitophagy inhibits amyloid-beta and tau pathology and reverses cognitive deficits in models of Alzheimer's disease. Nat Neurosci.

[13] Ye X, Sun X, Starovoytov V, Cai Q (2015). Parkin-mediated mitophagy in mutant hAPP neurons and Alzheimer’s disease patient brains. Hum Mol Genet.

[14] Martin-Maestro P, Gargini R, Perry G, Avila J, Garcia-Escudero V (2016). PARK2 enhancement is able to compensate mitophagy alterations found in sporadic Alzheimer’s disease. Hum Mol Genet.

[15] Du F, Yu Q, Yan S, Hu G, Lue LF, Walker DG, Wu L, Yan SF, Tieu K, Yan SS (2017). PINK1 signalling rescues amyloid pathology and mitochondrial dysfunction in Alzheimer’s disease. Brain.

[16] Cummins N, Tweedie A, Zuryn S, Bertran-Gonzalez J, Gotz J (2019). Disease-associated tau impairs mitophagy by inhibiting Parkin translocation to mitochondria. EMBO J.

[17] Goiran T, Duplan E, Chami M, Bourgeois A, El Manaa W, Rouland L, Dunys J, Lauritzen I, You H, Stambolic V (2018). Beta-amyloid precursor protein intracellular domain controls mitochondrial function by modulating phosphatase and tensin homolog-induced kinase 1 transcription in cells and in Alzheimer mice models. Biol Psychiatry.

[18] Barbero-Camps E, Roca-Agujetas V, Bartolessis I, de Dios C, Fernandez-Checa JC, Mari M, Morales A, Hartmann T, Colell A (2018). Cholesterol impairs autophagy-mediated clearance of amyloid beta while promoting its secretion. Autophagy.

[19] de Dios C, Bartolessis I, Roca-Agujetas V, Barbero-Camps E, Mari M, Morales A, Colell A (2019). Oxidative inactivation of amyloid beta-degrading proteases by cholesterol-enhanced mitochondrial stress. Redox Biol.

[20] Lazar AN, Bich C, Panchal M, Desbenoit N, Petit VW, Touboul D, Dauphinot L, Marquer C, Laprevote O, Brunelle A (2013). Time-of-flight secondary ion mass spectrometry (TOF-SIMS) imaging reveals cholesterol overload in the cerebral cortex of Alzheimer disease patients. Acta Neuropathol.

[21] Heverin M, Bogdanovic N, Lutjohann D, Bayer T, Pikuleva I, Bretillon L, Diczfalusy U, Winblad B, Bjorkhem I (2004). Changes in the levels of cerebral and extracerebral sterols in the brain of patients with Alzheimer's disease. J Lipid Res.

[22] Cutler RG, Kelly J, Storie K, Pedersen WA, Tammara A, Hatanpaa K, Troncoso JC, Mattson MP (2004). Involvement of oxidative stress-induced abnormalities in ceramide and cholesterol metabolism in brain aging and Alzheimer’s disease. Proc Natl Acad Sci U S A.

[23] Sun JH, Yu JT, Tan L (2015). The role of cholesterol metabolism in Alzheimer's disease. Mol Neurobiol.

[24] Jankowsky JL, Fadale DJ, Anderson J, Xu GM, Gonzales V, Jenkins NA, Copeland NG, Lee MK, Younkin LH, Wagner SL (2004). Mutant presenilins specifically elevate the levels of the 42 residue beta-amyloid peptide in vivo: evidence for augmentation of a 42-specific gamma secretase. Hum Mol Genet.

[25] Horton JD, Shimomura I, Brown MS, Hammer RE, Goldstein JL, Shimano H (1998). Activation of cholesterol synthesis in preference to fatty acid synthesis in liver and adipose tissue of transgenic mice overproducing sterol regulatory element-binding protein-2. J Clin Invest.

[26] Barbero-Camps E, Fernandez A, Martinez L, Fernandez-Checa JC, Colell A (2013). APP/PS1 mice overexpressing SREBP-2 exhibit combined Abeta accumulation and tau pathology underlying Alzheimer's disease. Hum Mol Genet.

[27] Fernandez A, Llacuna L, Fernandez-Checa JC, Colell A (2009). Mitochondrial cholesterol loading exacerbates amyloid beta peptide-induced inflammation and neurotoxicity. J Neurosci.

[28] Montine TJ, Phelps CH, Beach TG, Bigio EH, Cairns NJ, Dickson DW, Duyckaerts C, Frosch MP, Masliah E, Mirra SS (2012). National Institute on Aging-Alzheimer’s Association guidelines for the neuropathologic assessment of Alzheimer’s disease: a practical approach. Acta Neuropathol.

[29] Yu W, Gong JS, Ko M, Garver WS, Yanagisawa K, Michikawa M (2005). Altered cholesterol metabolism in niemann-pick type C1 mouse brains affects mitochondrial function. J Biol Chem.

[30] Diaz F, Barrientos A, Fontanesi F (2009). Evaluation of the mitochondrial respiratory chain and oxidative phosphorylation system using blue native gel electrophoresis. Curr Protoc Hum Genet.

[31] Katayama H, Kogure T, Mizushima N, Yoshimori T, Miyawaki A (2011). A sensitive and quantitative technique for detecting autophagic events based on lysosomal delivery. Chem Biol.

[32] Podlesniy P, Puigros M, Serra N, Fernandez-Santiago R, Ezquerra M, Tolosa E, Trullas R (2019). Accumulation of mitochondrial 7S DNA in idiopathic and LRRK2 associated Parkinson’s disease. EBioMedicine.

[33] Sun N, Malide D, Liu J, Rovira II, Combs CA, Finkel T (2017). A fluorescence-based imaging method to measure in vitro and in vivo mitophagy using mt-Keima. Nat Protoc.

[34] Schindelin J, Arganda-Carreras I, Frise E, Kaynig V, Longair M, Pietzsch T, Preibisch S, Rueden C, Saalfeld S, Schmid B (2012). Fiji: an open-source platform for biological-image analysis. Nat Methods.

[35] Kwiatkowska K, Marszalek-Sadowska E, Traczyk G, Koprowski P, Musielak M, Lugowska A, Kulma M, Grzelczyk A, Sobota A (2014). Visualization of cholesterol deposits in lysosomes of Niemann-pick type C fibroblasts using recombinant perfringolysin O. Orphanet J Rare Dis.

[36] Podlesniy P, Trullas R (2017). Absolute measurement of gene transcripts with Selfie-digital PCR. Sci Rep.

[37] Marzella L, Ahlberg J, Glaumann H (1982). Isolation of autophagic vacuoles from rat liver: morphological and biochemical characterization. J Cell Biol.

[38] Van Laar VS, Arnold B, Cassady SJ, Chu CT, Burton EA, Berman SB (2011). Bioenergetics of neurons inhibit the translocation response of Parkin following rapid mitochondrial depolarization. Hum Mol Genet.

[39] Wang X, Su B, Lee HG, Li X, Perry G, Smith MA, Zhu X (2009). Impaired balance of mitochondrial fission and fusion in Alzheimer’s disease. J Neurosci.

[40] Scherz-Shouval R, Shvets E, Fass E, Shorer H, Gil L, Elazar Z. Reactive oxygen species are essential for autophagy and specifically regulate the activity of Atg4. EMBO J. 2007;26:1749-60. 10.1038/sj.emboj.7601623 PMC1847657 17347651

[41] Amemiya-Kudo M, Shimano H, Hasty AH, Yahagi N, Yoshikawa T, Matsuzaka T, Okazaki H, Tamura Y, Iizuka Y, Ohashi K (2002). Transcriptional activities of nuclear SREBP-1a, −1c, and −2 to different target promoters of lipogenic and cholesterogenic genes. J Lipid Res.

[42] Itakura E, Kishi-Itakura C, Koyama-Honda I, Mizushima N (2012). Structures containing Atg9A and the ULK1 complex independently target depolarized mitochondria at initial stages of Parkin-mediated mitophagy. J Cell Sci.

[43] D'Erchia AM, Atlante A, Gadaleta G, Pavesi G, Chiara M, De Virgilio C, Manzari C, Mastropasqua F, Prazzoli GM, Picardi E (2015). Tissue-specific mtDNA abundance from exome data and its correlation with mitochondrial transcription, mass and respiratory activity. Mitochondrion.

[44] Wenz T (2013). Regulation of mitochondrial biogenesis and PGC-1alpha under cellular stress. Mitochondrion.

[45] Austin S, St-Pierre J (2012). PGC1alpha and mitochondrial metabolism--emerging concepts and relevance in ageing and neurodegenerative disorders. J Cell Sci.

[46] Meissner C, Lorenz H, Hehn B, Lemberg MK (2015). Intramembrane protease PARL defines a negative regulator of PINK1- and PARK2/Parkin-dependent mitophagy. Autophagy.

[47] Shi G, McQuibban GA (2017). The mitochondrial rhomboid protease PARL is regulated by PDK2 to integrate mitochondrial quality control and metabolism. Cell Rep.

[48] Kinoshita E, Kinoshita-Kikuta E, Takiyama K, Koike T (2006). Phosphate-binding tag, a new tool to visualize phosphorylated proteins. Mol Cell Proteomics.

[49] van Wijk SJ, Fiskin E, Putyrski M, Pampaloni F, Hou J, Wild P, Kensche T, Grecco HE, Bastiaens P, Dikic I (2012). Fluorescence-based sensors to monitor localization and functions of linear and K63-linked ubiquitin chains in cells. Mol Cell.

[50] Heo JM, Ordureau A, Paulo JA, Rinehart J, Harper JW (2015). The PINK1-PARKIN mitochondrial Ubiquitylation pathway drives a program of OPTN/NDP52 recruitment and TBK1 activation to promote Mitophagy. Mol Cell.

[51] Narendra D, Kane LA, Hauser DN, Fearnley IM, Youle RJ (2010). p62/SQSTM1 is required for Parkin-induced mitochondrial clustering but not mitophagy; VDAC1 is dispensable for both. Autophagy.

[52] Lazarou M, Sliter DA, Kane LA, Sarraf SA, Wang C, Burman JL, Sideris DP, Fogel AI, Youle RJ (2015). The ubiquitin kinase PINK1 recruits autophagy receptors to induce mitophagy. Nature.

[53] Richter B, Sliter DA, Herhaus L, Stolz A, Wang C, Beli P, Zaffagnini G, Wild P, Martens S, Wagner SA (2016). Phosphorylation of OPTN by TBK1 enhances its binding to Ub chains and promotes selective autophagy of damaged mitochondria. Proc Natl Acad Sci U S A.

[54] Slowicka K, Vereecke L, van Loo G (2016). Cellular functions of Optineurin in health and disease. Trends Immunol.

[55] Osawa T, Mizuno Y, Fujita Y, Takatama M, Nakazato Y, Okamoto K (2011). Optineurin in neurodegenerative diseases. Neuropathology.

[56] Gao J, Ohtsubo M, Hotta Y, Minoshima S (2014). Oligomerization of optineurin and its oxidative stress- or E50K mutation-driven covalent cross-linking: possible relationship with glaucoma pathology. PLoS One.

[57] Mao J, Xia Q, Liu C, Ying Z, Wang H, Wang G (2017). A critical role of Hrd1 in the regulation of optineurin degradation and aggresome formation. Hum Mol Genet.

[58] Tan JM, Wong ES, Kirkpatrick DS, Pletnikova O, Ko HS, Tay SP, Ho MW, Troncoso J, Gygi SP, Lee MK (2008). Lysine 63-linked ubiquitination promotes the formation and autophagic clearance of protein inclusions associated with neurodegenerative diseases. Hum Mol Genet.

[59] Kawaguchi Y, Kovacs JJ, McLaurin A, Vance JM, Ito A, Yao TP (2003). The deacetylase HDAC6 regulates aggresome formation and cell viability in response to misfolded protein stress. Cell.

[60] Kim KY, Stevens MV, Akter MH, Rusk SE, Huang RJ, Cohen A, Noguchi A, Springer D, Bocharov AV, Eggerman TL (2011). Parkin is a lipid-responsive regulator of fat uptake in mice and mutant human cells. J Clin Invest.

[61] Ivatt RM, Sanchez-Martinez A, Godena VK, Brown S, Ziviani E, Whitworth AJ (2014). Genome-wide RNAi screen identifies the Parkinson disease GWAS risk locus SREBF1 as a regulator of mitophagy. Proc Natl Acad Sci U S A.

[62] Bingol B, Tea JS, Phu L, Reichelt M, Bakalarski CE, Song Q, Foreman O, Kirkpatrick DS, Sheng M (2014). The mitochondrial deubiquitinase USP30 opposes parkin-mediated mitophagy. Nature.

[63] Ashrafi G, Schlehe JS, LaVoie MJ, Schwarz TL (2014). Mitophagy of damaged mitochondria occurs locally in distal neuronal axons and requires PINK1 and Parkin. J Cell Biol.

[64] McWilliams TG, Prescott AR, Montava-Garriga L, Ball G, Singh F, Barini E, Muqit MMK, Brooks SP, Ganley IG (2018). Basal Mitophagy occurs independently of PINK1 in mouse tissues of high metabolic demand. Cell Metab.

[65] Palacino JJ, Sagi D, Goldberg MS, Krauss S, Motz C, Wacker M, Klose J, Shen J (2004). Mitochondrial dysfunction and oxidative damage in parkin-deficient mice. J Biol Chem.

[66] Gautier CA, Kitada T, Shen J (2008). Loss of PINK1 causes mitochondrial functional defects and increased sensitivity to oxidative stress. Proc Natl Acad Sci U S A.

[67] Pickrell AM, Huang CH, Kennedy SR, Ordureau A, Sideris DP, Hoekstra JG, Harper JW, Youle RJ (2015). Endogenous Parkin preserves dopaminergic substantia nigral neurons following mitochondrial DNA mutagenic stress. Neuron.

[68] Wang Y, Nartiss Y, Steipe B, McQuibban GA, Kim PK (2012). ROS-induced mitochondrial depolarization initiates PARK2/PARKIN-dependent mitochondrial degradation by autophagy. Autophagy.

[69] Xiao B, Deng X, Lim GGY, Xie S, Zhou ZD, Lim KL, Tan EK (2017). Superoxide drives progression of Parkin/PINK1-dependent mitophagy following translocation of Parkin to mitochondria. Cell Death Dis.

[70] Cai Q, Zakaria HM, Simone A, Sheng ZH (2012). Spatial parkin translocation and degradation of damaged mitochondria via mitophagy in live cortical neurons. Curr Biol.

[71] Evans CS, Holzbaur EL. Degradation of engulfed mitochondria is rate-limiting in optineurin-mediated mitophagy in neurons. ELife. 2020;9:e50260. 10.7554/eLife.50260 PMC6959996 31934852

[72] Shin YS, Ryall JG, Britto JM, Lau CL, Devenish RJ, Nagley P, Beart PM (2019). Inhibition of bioenergetics provides novel insights into recruitment of PINK1-dependent neuronal mitophagy. J Neurochem.

[73] Jin SM, Youle RJ (2013). The accumulation of misfolded proteins in the mitochondrial matrix is sensed by PINK1 to induce PARK2/Parkin-mediated mitophagy of polarized mitochondria. Autophagy.

[74] Cenini G, Rub C, Bruderek M, Voos W (2016). Amyloid beta-peptides interfere with mitochondrial preprotein import competence by a coaggregation process. Mol Biol Cell.

[75] An YA, Crewe C, Asterholm IW, Sun K, Chen S, Zhang F, Shao M, Funcke JB, Zhang Z, Straub L (2019). Dysregulation of amyloid precursor protein impairs adipose tissue mitochondrial function and promotes obesity. Nat Metab.

[76] Bertolin G, Ferrando-Miguel R, Jacoupy M, Traver S, Grenier K, Greene AW, Dauphin A, Waharte F, Bayot A, Salamero J (2013). The TOMM machinery is a molecular switch in PINK1 and PARK2/PARKIN-dependent mitochondrial clearance. Autophagy.

[77] Shi G, Lee JR, Grimes DA, Racacho L, Ye D, Yang H, Ross OA, Farrer M, McQuibban GA, Bulman DE (2011). Functional alteration of PARL contributes to mitochondrial dysregulation in Parkinson’s disease. Hum Mol Genet.

[78] Ordonez MP, Roberts EA, Kidwell CU, Yuan SH, Plaisted WC, Goldstein LS (2012). Disruption and therapeutic rescue of autophagy in a human neuronal model of Niemann pick type C1. Hum Mol Genet.

[79] Sun N, Yun J, Liu J, Malide D, Liu C, Rovira II, Holmstrom KM, Fergusson MM, Yoo YH, Combs CA (2015). Measuring in vivo mitophagy. Mol Cell.

[80] Singhal A, Krystofiak ES, Jerome WG, Song B (2020). 2-Hydroxypropyl-gamma-cyclodextrin overcomes NPC1 deficiency by enhancing lysosome-ER association and autophagy. Sci Rep.

[81] Wei W, Keogh MJ, Wilson I, Coxhead J, Ryan S, Rollinson S, Griffin H, Kurzawa-Akanbi M, Santibanez-Koref M, Talbot K (2017). Mitochondrial DNA point mutations and relative copy number in 1363 disease and control human brains. Acta Neuropathol Commun.

[82] Coskun PE, Beal MF, Wallace DC (2004). Alzheimer's brains harbor somatic mtDNA control-region mutations that suppress mitochondrial transcription and replication. Proc Natl Acad Sci U S A.

[83] Thubron EB, Rosa HS, Hodges A, Sivaprasad S, Francis PT, Pienaar IS, Malik AN (2019). Regional mitochondrial DNA and cell-type changes in post-mortem brains of non-diabetic Alzheimer’s disease are not present in diabetic Alzheimer’s disease. Sci Rep.

[84] Hoenig MR, Sellke FW (2010). Insulin resistance is associated with increased cholesterol synthesis, decreased cholesterol absorption and enhanced lipid response to statin therapy. Atherosclerosis.

[85] Guerrero-Gomez D, Mora-Lorca JA, Saenz-Narciso B, Naranjo-Galindo FJ, Munoz-Lobato F, Parrado-Fernandez C, Goikolea J, Cedazo-Minguez A, Link CD, Neri C (2019). Loss of glutathione redox homeostasis impairs proteostasis by inhibiting autophagy-dependent protein degradation. Cell Death Differ.

[86] Yan J, Seibenhener ML, Calderilla-Barbosa L, Diaz-Meco MT, Moscat J, Jiang J, Wooten MW, Wooten MC (2013). SQSTM1/p62 interacts with HDAC6 and regulates deacetylase activity. PLoS One.

[87] Simoes-Pires C, Zwick V, Nurisso A, Schenker E, Carrupt PA, Cuendet M (2013). HDAC6 as a target for neurodegenerative diseases: what makes it different from the other HDACs?. Mol Neurodegener.

[88] Ito Y, Ofengeim D, Najafov A, Das S, Saberi S, Li Y, Hitomi J, Zhu H, Chen H, Mayo L (2016). RIPK1 mediates axonal degeneration by promoting inflammation and necroptosis in ALS. Science.

[89] Arenas F, Castro F, Nunez S, Gay G, Garcia-Ruiz C, Fernandez-Checa JC (2020). STARD1 and NPC1 expression as pathological markers associated with astrogliosis in post-mortem brains from patients with Alzheimer's disease and Down syndrome. Aging.

[90] Webber KM, Stocco DM, Casadesus G, Bowen RL, Atwood CS, Previll LA, Harris PL, Zhu X, Perry G, Smith MA (2006). Steroidogenic acute regulatory protein (StAR): evidence of gonadotropin-induced steroidogenesis in Alzheimer disease. Mol Neurodegener.

[91] Barbero-Camps E, Fernandez A, Baulies A, Martinez L, Fernandez-Checa JC, Colell A (2014). Endoplasmic reticulum stress mediates amyloid beta neurotoxicity via mitochondrial cholesterol trafficking. Am J Pathol.

