# Supplementary material for: Cholesterol alters mitophagy by impairing optineurin recruitment and lysosomal clearance in Alzheimer’s disease
Source: Mol Neurodegener. 2021 Mar 8;16:15. doi: 10.1186/s13024-021-00435-6 (PMC7941983; doi:10.1186/s13024-021-00435-6)
Supplement: Supplementary file 1 — Additional file 1: Figure S1. Upregulated mRNA expression levels of cholesterol-related genes in hippocampus from mice that overexpress SREBF2. Figure S2. Cross-contamination analysis of autophagosomal (F1) and lysosomal (F3) fractions isolated by density gradient centrifugation. Figure S3. Cholesterol enrichment in SH-SY5Y cells prevents mitochondrial lysosomal clearance after Aβ induction of mitophagy. Figure S4. Impaired mitophagy flux in cholesterol-enriched cells incubated with CCCP. Figure S5. Cell Viability of WT and SREBF2 neurons was not affected by the treatment with Aβ, rapamycin or GSH ethyl ester (GSHee). Figure S6. Defective mitophagy in SREBF2 neurons exposed to rapamycin. Figure S7. Mitochondrial membrane potential remains unchanged in cultured primary neurons after Aβ exposure. Figure S8. SREBF2 overexpression in APP-PSEN1 neurons results in an accumulation of mitophagosomes but prevents mitophagy completion. Figure S9. Immunoblot analysis of LC3B levels in mitochondria isolated from brains of WT and APP-PSEN1-SREBF2 mice from 4, 7, and 10 months of age. Figure S10. Quantification of mitochondrial DNA (mtDNA) in the prefrontal cortex (PFC), hippocampus (HP), and cerebellum (CB) from APP-PSEN1-SREBF2 mice analyzed by range of age. Figure S11. Mitochondria from the brains of 7-month-old APP-PSEN1-SREBF2 mice display enriched content of K63 polyubiquitinated proteins. Figure S12. Differential recruitment of autophagy receptors in brains of APP-PSEN1-SREBF2 mice and in cholesterol-enriched SH-SY5Y cells exposed to Aβ. Figure S13. Appearance of OPTN-positive aggregates in CA3-CA2 hippocampal layers concomitant to the neuropathological AD progression. Figure S14. Selective recognition of cholesterol by GST-PFO. Figure S15. Differential cholesterol distribution in hippocampal neurons with the progression of neuropathological AD stages. Figure S16. Uncropped scans of western blots included in Fig. 1. Figure S17. Uncropped scans of western blots included in [file 13024_2021_435_MOESM1_ESM.docx]

**supplementary data**

**CHOLESTEROL ALTERS MITOPHAGY BY IMPAIRING OPTINEURIN RECRUITMENT AND LYSOSOMAL CLEARANCE IN ALZHEIMER’S DISEASE**

Vicente Roca-Agujetas^1,3^, Elisabet Barbero-Camps^1^, Cristina de Dios^1,3,4^, Petar Podlesniy^1,2,3^, Xenia Abadin^1^, Albert Morales^1^, Montserrat Marí^1^, Ramon Trullàs^1,2,3^, Anna Colell^1, 3^

^1^Department of Cell Death and Proliferation, and ^2^Neurobiology Unit, Institut d'Investigacions Biomèdiques de Barcelona (IIBB), Consejo Superior de Investigaciones Científicas (CSIC). Institut d'Investigacions Biomèdiques August Pi i Sunyer (IDIBAPS), Barcelona, Spain.

^3^Centro de Investigación Biomédica en Red sobre Enfermedades Neurodegenerativas (CIBERNED), Spain.

^4^Departament de Biomedicina, Facultat de Medicina, Universitat de Barcelona, Barcelona, Spain.

**SUPPLEMENTARY FIGURES**

**
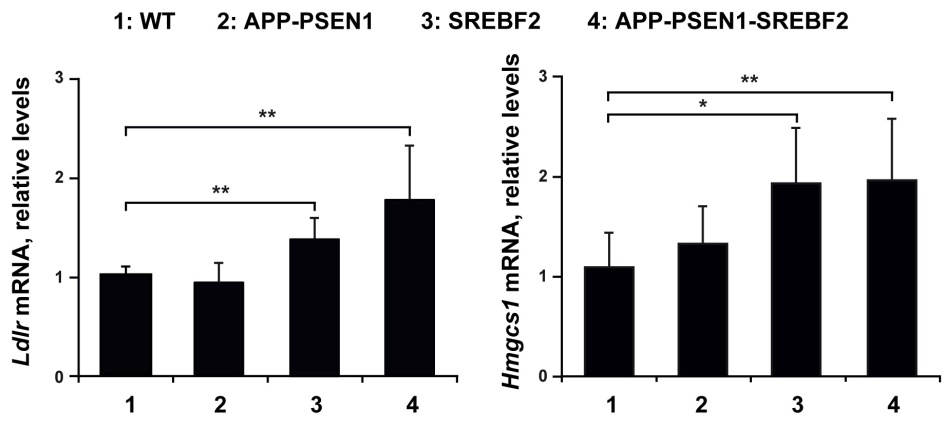
**

**Figure 1.** **Upregulated mRNA expression levels of cholesterol-related genes in hippocampus from mice that overexpress SREBF2.** mRNA levels of LDL receptor and HMG-CoA synthase in brains from WT (wild-type), SREBF2, APP-PSEN1, and APP-PSEN1-SREBF2 mice (n = 3). Student’s t-test. **P* < 0.05, ***P* < 0.01 (data are mean ± SD).


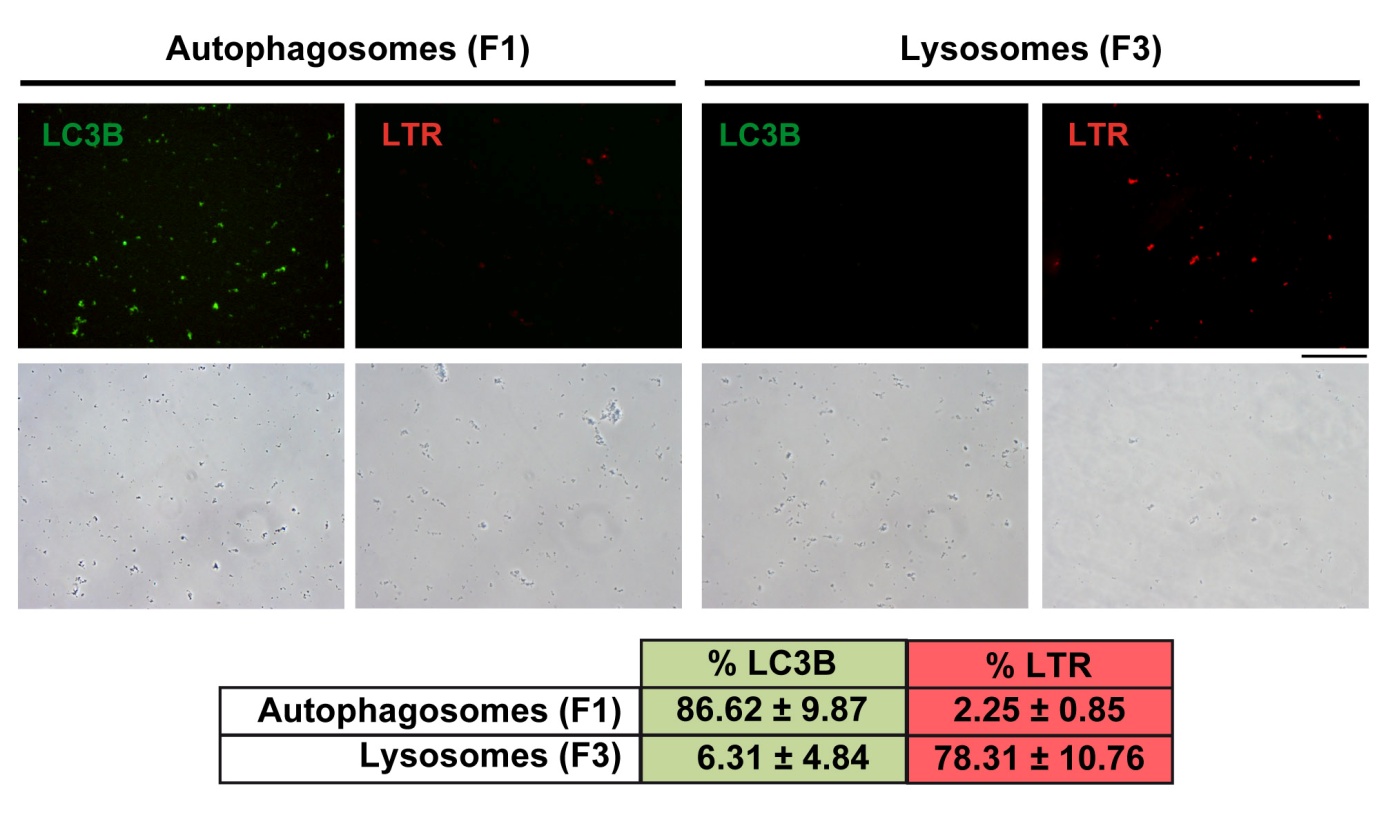


**Figure 2. Cross-contamination analysis of autophagosomal (F1) and lysosomal (F3) fractions isolated** **by density gradient centrifugation.** Representative images of autophagosomes and lysosomes isolated from APP-PSEN1-SREBF2 brains. Fractions were individually labeled with anti-LC3 antibody plus FITC-labeled secondary antibody and LysoTracker Red. The number of particles for each fluorophore was quantified and divided by the total count from its corresponding phase-contrast images. Values are expressed as a percentage of positively stained particles per microscopic field. Counts are from 10 different fields. Scale bar: 15 μm.


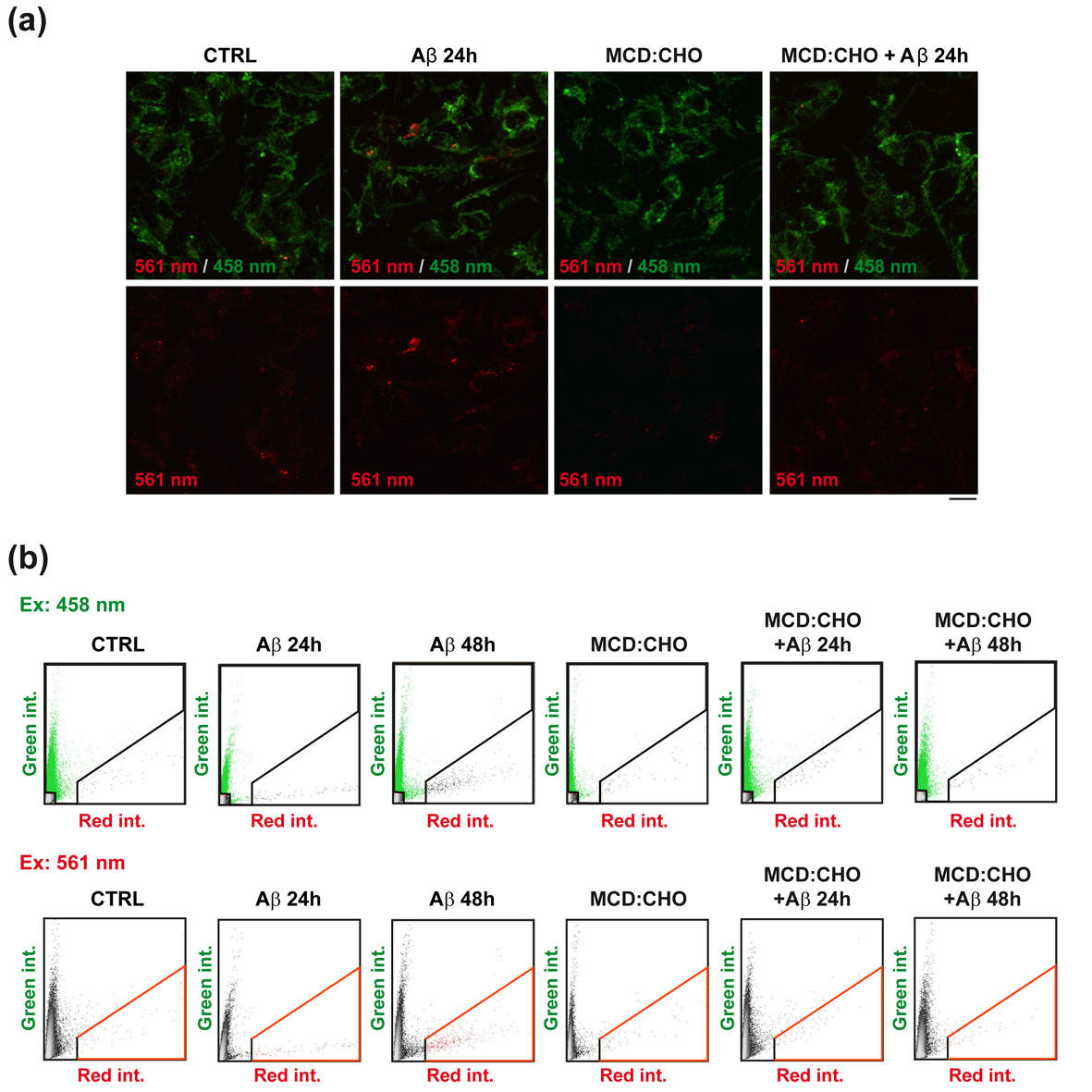


**Figure 3.** **Cholesterol enrichment in SH-SY5Y cells prevents mitochondrial lysosomal clearance after Aβ induction of mitophagy.** Cells were incubated with a complex of cholesterol:methyl-β-cyclodextrin (CHO:MCD) containing 50 μg/ml cholesterol for 1 h followed by 4h of recovery. Mitophagy was induced with Aβ (10 μM) for 24 h or 48 h. **(a)** Dual-excitation ratiometric imaging of control and cholesterol-enriched cells stably expressing mt-mKeima after Aβ exposure. The emission signal obtained after excitation with the 458 nm laser is shown in green, and that obtained after excitation with the 561 nm laser is shown in red. Scale bars: 25 μm. **(b)** Intensity scatter plots. For each condition, all the pixels from the sum projection of green and red channels are represented. Selected red areas are the pixels with a ratio of red/green intensity higher than 1.5. Mitophagy index is calculated by dividing the pixels from the red area by the total sum of red and green selection, after subtracting the background.


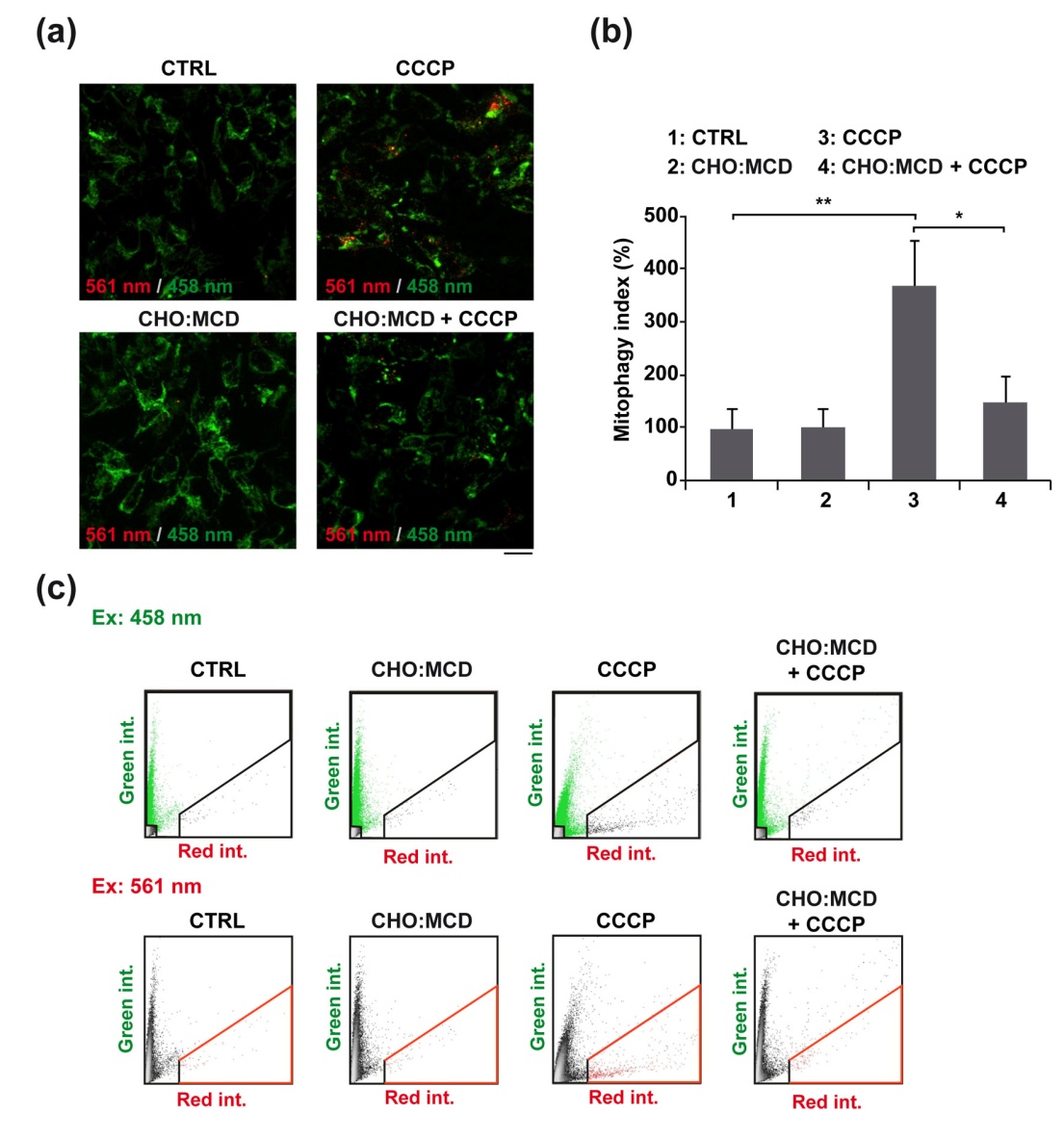


**Figure 4.** **Impaired** **mitophagy flux in cholesterol-enriched cells incubated with CCCP.** Cells were first incubated with a complex of cholesterol:methyl-β-cyclodextrin (CHO:MCD) containing 50 μg/ml cholesterol for 1h followed by 4h of recovery. Then, mitophagy was induced with CCCP (10 μM) for 24 h. **(a)** Dual-excitation ratiometric imaging of control and cholesterol-enriched cells stably expressing mt-mKeima after CCCP exposure. Scale bars: 25 μm. **(b)** Mitophagy index calculated by dividing the pixels from the red area in the intensity scatter plots by the total sum of red and green selection, after subtracting the background. **(c)** Intensity scatter plots. For each condition, all the pixels from the sum projection of green and red channels are represented. Selected red areas are the pixels with a ratio of red/green intensity higher than 1.5. The mitophagy index is expressed as percentage of control (n = 3). One-way ANOVA. **P* < 0.05; ***P* < 0.01 (data are mean ± SD).


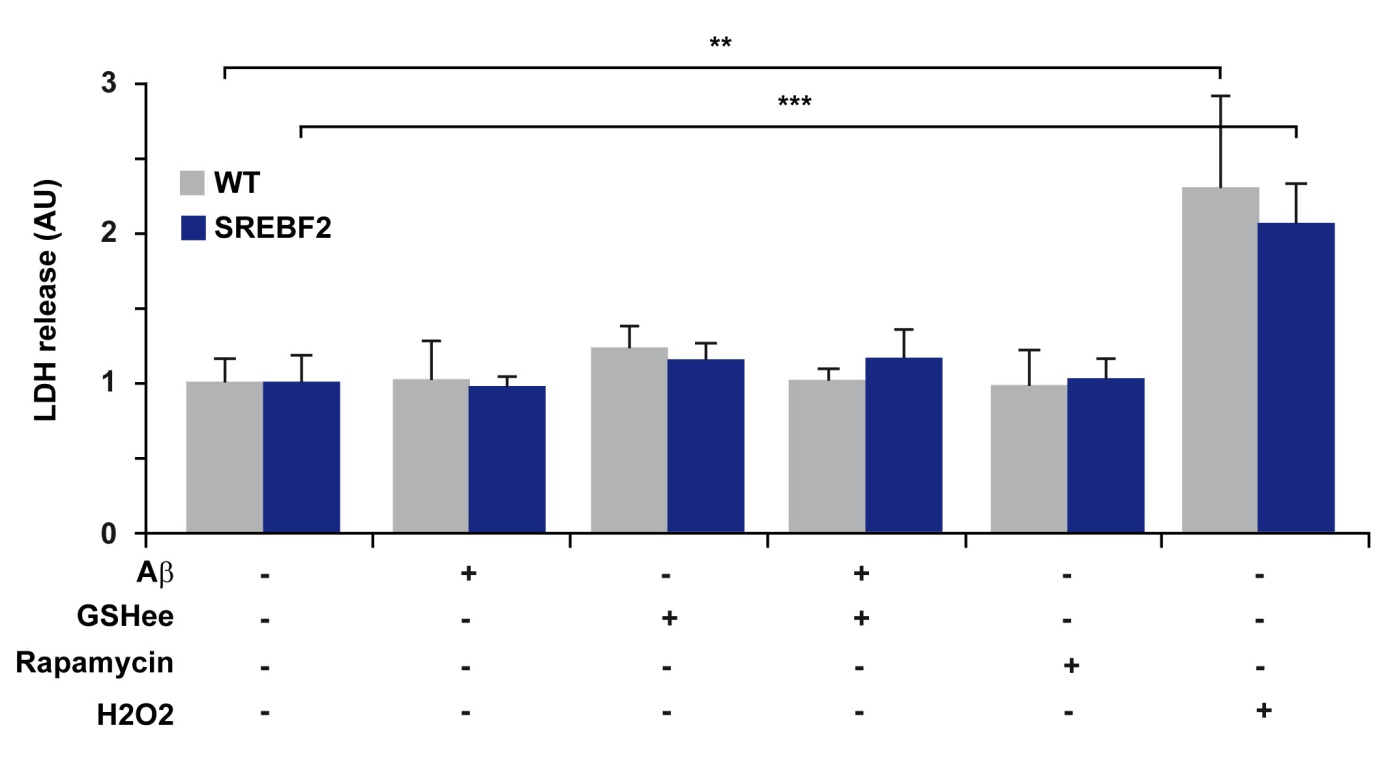


**Figure 5. Cell Viability of WT and SREBF2 neurons was not affected by the treatment with Aβ, rapamycin or GSH ethyl ester (GSHee).** Embryonic cortical and hippocampal neurons isolated from WT and SREBF2 mice were incubated with Aβ (5uM) or rapamycin (10 nM) for 24 h with or without GSHee pre-incubation (0.5 mM, for 30 min). Cytotoxicity of the different compounds was assessed by lactate dehydrogenase (LDH) assay and expressed as % of LDH release (n = 3). H2O2 (300 μM for 24 h) treatment was used as a positive control. Student’s t-test. ***P* < 0.01, ****P* < 0.001 (data are mean ± SD). AU: arbitrary units.


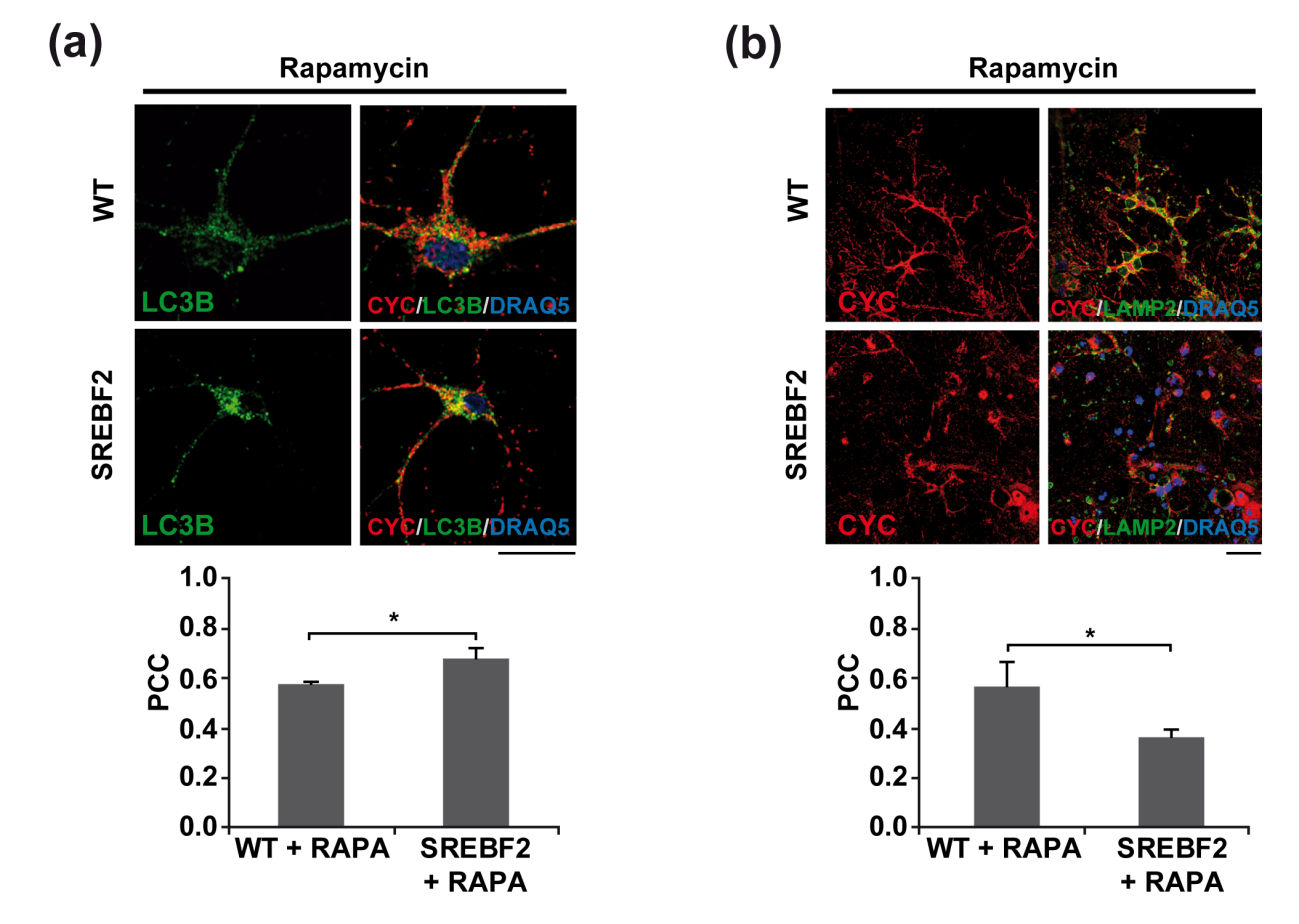


**Figure 6. Defective mitophagy in SREBF2 neurons exposed to rapamycin.** Embryonic cortical and hippocampal neurons isolated from WT and SREBF2 mice were incubated with rapamycin (RAPA, 10 nM) for 24h. **(a)** Shown are representative confocal images of a double immunofluorescence for LC3B (green) and CYC (red). **(b)** Representative confocal images of the immunostaining for LAMP2 (green) and CYC (red). The Pearson’s correlation coefficient (PCC) was used as a measured of colocalization of Alexa fluor 488 (LC3B, LAMP2) with Cy3 (CYC) signals and calculated from 3 independent experiments (at least 3 random fields were analyzed per condition). Scale bars: 25 μm. **P* < 0.05 (data are mean ± SD).


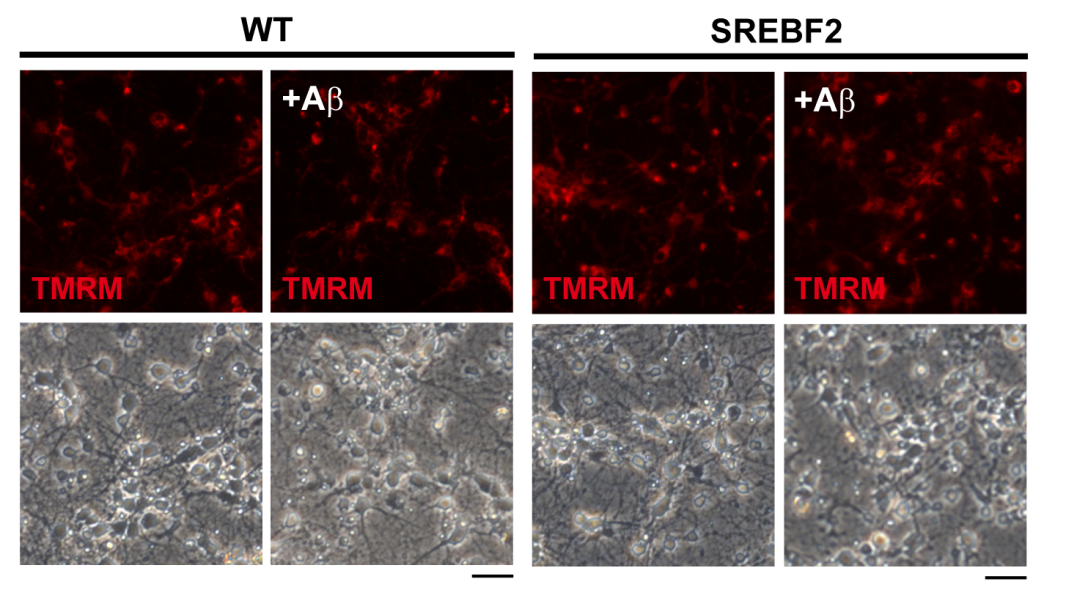


**Figure 7. Mitochondrial membrane potential remains unchanged in cultured primary neurons after Aβ exposure.**  Embryonic cortical/hippocampal neurons isolated from WT and SREBF2 mice were incubated with Aβ (5 μM) for 24 h and stained with TMRM (1 nM) for 30 min. Shown are representative fluorescence microscopy images of TMRM accompanied by the corresponding phase-contrast images (n = 3). Scale bars: 25 μm.


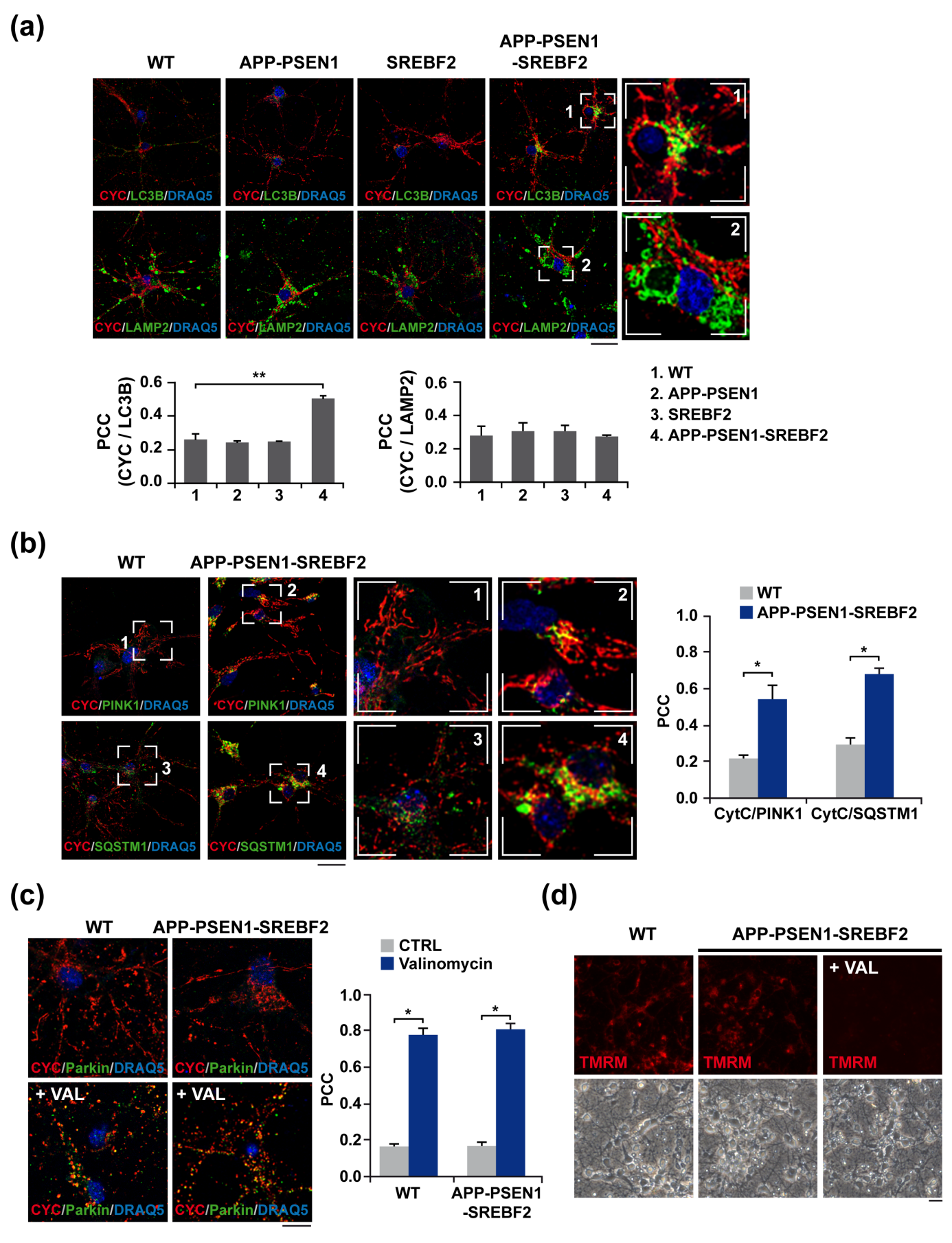


**Figure 8. SREBF2 overexpression in APP-PSEN1 neurons results in an accumulation of mitophagosomes but prevents mitophagy completion.** **(a and b)** Embryonic cortical/hippocampal neurons were isolated from WT (wild-type), SREBF2, APP-PSEN1 and APP-PSEN1-SREBF2 mice. **(a)** Shown are representative confocal images of a double immunofluorescence for LC3B (green) and CYC (red) and for LAMP2 (green) and CYC (red). Scale bar: 25 μm. **(b)** Representative confocal images of double immunostainings for PINK1 (green) and CYC (red) and for SQSTM1 (green) and CYC (red). Scale bar: 25 μm. **(c and d)** Neuronal cultures were incubated with valinomycin (10 μM) for 3 h. **(c)** Shown are representative confocal images of a double immunofluorescence for CYC (red) and parkin (green). Scale bars: 10 μm. **(d)** After valinomycin incubation cells were stained with TMRM (1 nM) for 30 min. Shown are representative fluorescence microscopy images of TMRM accompanied by the corresponding phase-contrast images. Scale bars: 25 μm. Nuclei were counterstained with DRAQ5 (blue). Insets show a 3-fold magnification of the indicated regions. In all the cases, the Pearson’s correlation coefficient (PCC) was calculated from 3 independent experiments (at least 6 random fields were analyzed per condition). **P* < 0.05; ***P* < 0.01 (data are mean ± SD).


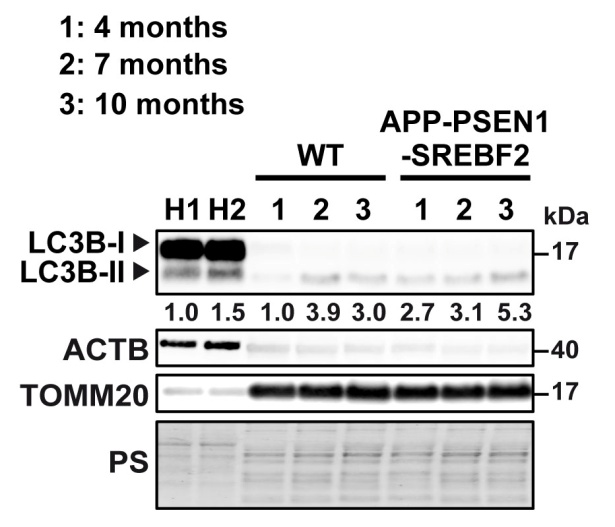


**Figure 9. Immunoblot analysis of LC3B levels in mitochondria isolated from brains of WT and APP-PSEN1-SREBF2 mice from 4, 7, and 10 months of age.** All densitometry values were first normalized to Ponceau S (PS) staining to adjust for protein loading. Then, LC3B-II values were normalized to the values of the corresponding ACTB/actin β (homogenates, H) or TOMM20 (mitochondria) bands. H1: homogenate from 9-month-old WT mouse. H2: homogenate from 9-month-old APP-PSEN1-SREBF2 mouse.

**
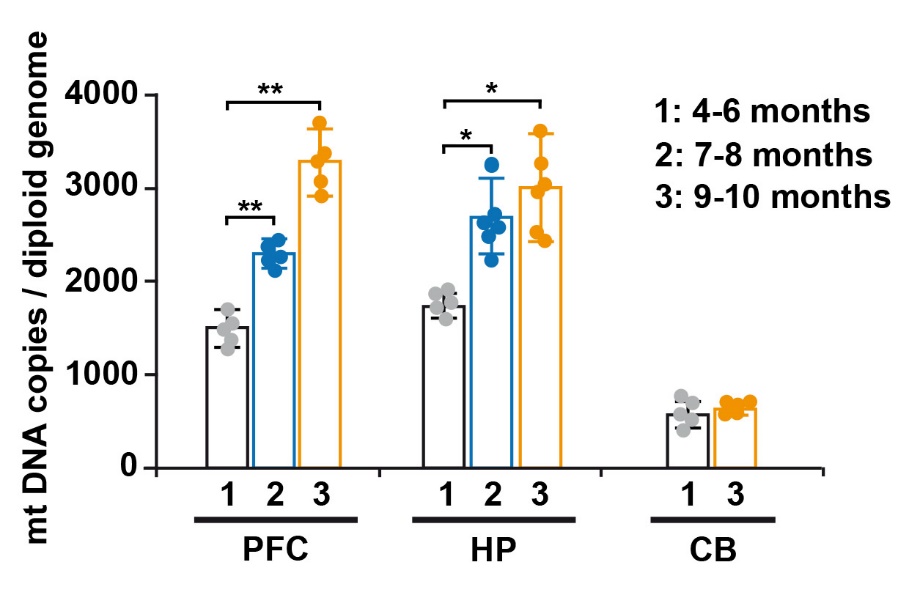
**

**Figure 10. Quantification of mitochondrial DNA (mtDNA) in the prefrontal cortex (PFC), hippocampus (HP), and cerebellum (CB) from APP-PSEN1-SREBF2 mice analyzed by range of age.** mtDNA copy numbers were normalized to the copy number of *Bax* and *Gsk3β* genes as a mean of total diploid genome (*n* = 5-6 per range of age and genotype). One-way ANOVA. **P* < 0.05; ***P* < 0.01 (data are mean ± SD).

**
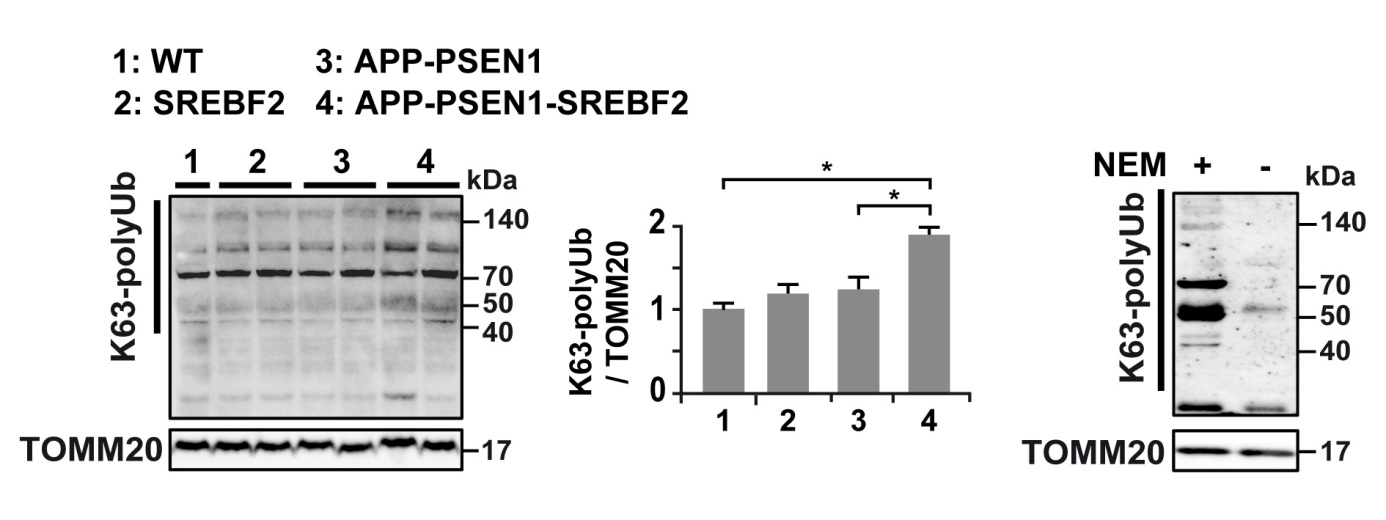
**

**Figure 11. Mitochondria from the brains of 7-month-old APP-PSEN1-SREBF2 mice display enriched content of K63 polyubiquitinated proteins.** K63-linkage specific polyubiquitin antibodies were used to detect K63 ubiquitination of proteins in samples. During isolation, deubiquitination of the proteins was prevented by including 10 mM N-ethylmaleimide (NEM) in the isolation buffer. As a negative control, samples were incubated without NEM for 1h at 4ºC before loading. Densitometry of the bands corresponding to K63 polyubiquitin immunoreactivity was normalized to TOMM20 values (n = 3). Student’s t-test. **P* < 0.05 (data are mean ± SD).

**
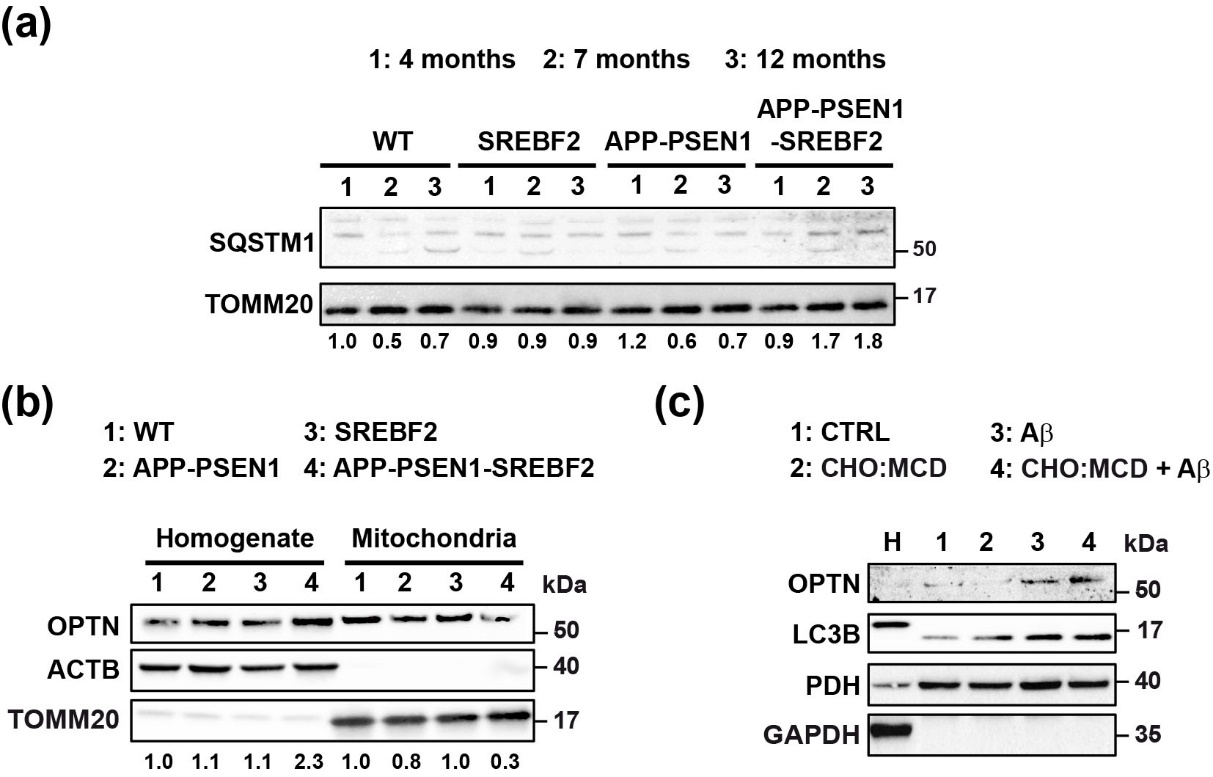
**

**Figure 12. Differential recruitment of autophagy receptors in brains of APP-PSEN1-SREBF2 mice and in cholesterol-enriched SH-SY5Y cells exposed to Aβ. (a)** Western blot analysis of SQSTM1 levels in the mitochondria-rich fraction from brains of WT and the indicated transgenic mice at 4, 7 and 12 month of age. **(b)** Western blot analysis of OPTN in brain homogenates and isolated mitochondria from 8-month-old WT and the indicated transgenic mice. Densitometric values of the SQSTM1 and OPTN bands were normalized to the values of the corresponding ACTB/actin β (homogenates) and TOMM20 (mitochondrial fraction) bands. **(b)** Immunoblot analysis of OPTN and LC3B levels in the mitochondria-rich fraction of SH-SY5Y cells. Cells were incubated with a complex of cholesterol:methyl-β-cyclodextrin (CHO:MCD) containing 50 μg/ml cholesterol during 1 h followed by 4 h of recovery. After cholesterol-enrichment, mitophagy was induced with Aβ (10 μM) for 24h. H: homogenate from control cells. GAPDH and PDH levels were assessed as homogenate and mitochondria markers, respectively.


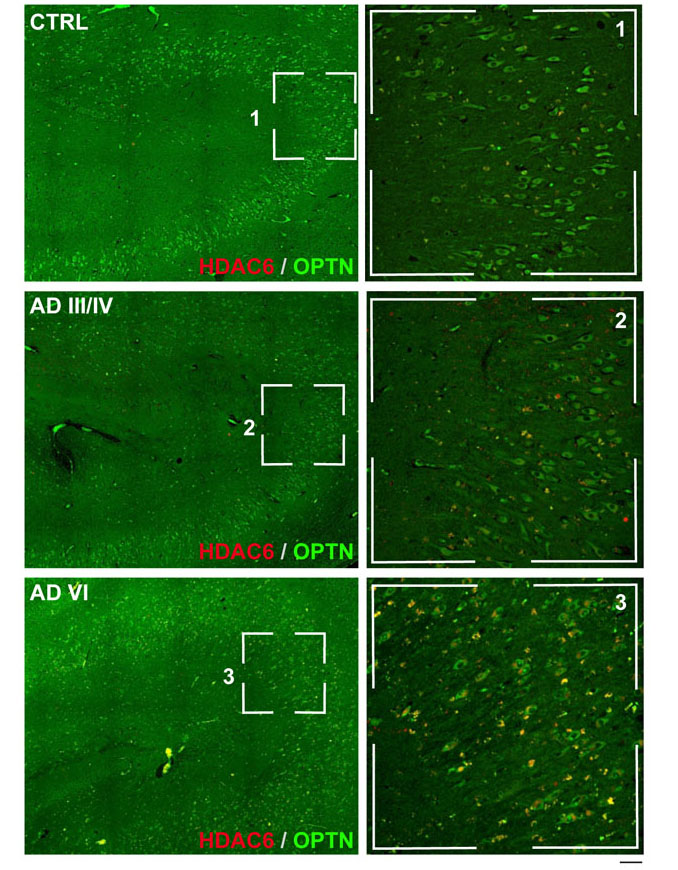


**Figure 13. Appearance of OPTN-positive aggregates in CA3-CA2 hippocampal layers concomitant to the neuropathological AD progression.** Hippocampal slices from control (CTRL) and AD patients classified into three groups according to their neuropathological hallmarks and following the “ABC” score: CTRL, intermediate AD (AD III-IV) and high AD (AD VI). Shown are representative stitched confocal photomicrograph of the hippocampal CA3-CA2 layers with double immunofluorescence for OPTN (green) and HDAC6 (red). Magnifications are sum slices projections of the indicated region. Scale bar: 50 μm.


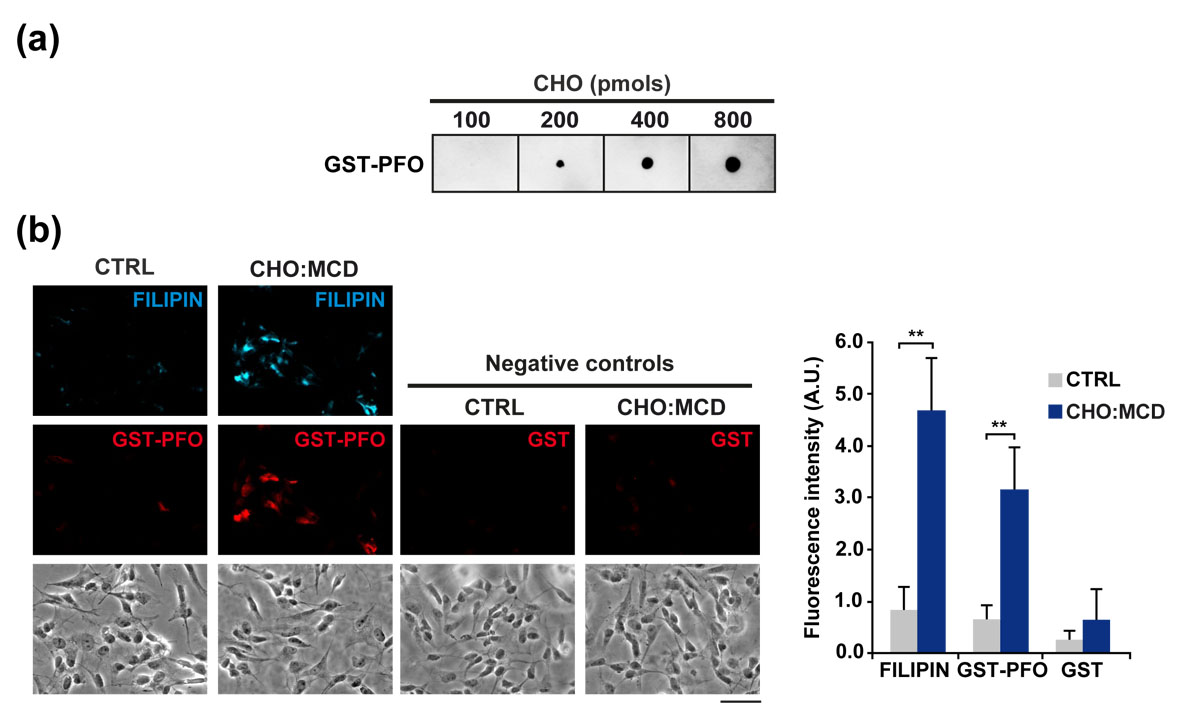


**Figure 14. Selective recognition of cholesterol by GST-PFO. (a)** Protein-lipid overlay assay. Indicated amounts of cholesterol were spotted in a nitrocellulose membrane, incubated with GST-PFO (2 μg/ml) for 1h and immunoblotted. **(b)** SH-SY5Y cells were incubated with a complex of cholesterol:methyl-β-cyclodextrin (CHO:MCD) containing 50 μg/ml cholesterol during 1 h. Then, cells were fixed and incubated with GST-PFO (20 μg/ml) and filipin (0.25 mg/ml) for 45 min. Negative controls without GST-PFO incubation were included. Fluorescence intensity was expressed as the integrated density of the fluorescence signal from each image (n = 3). Scale bar: 50 μm. Student’s t-test. ***P* < 0.01 (data are mean ± SD).


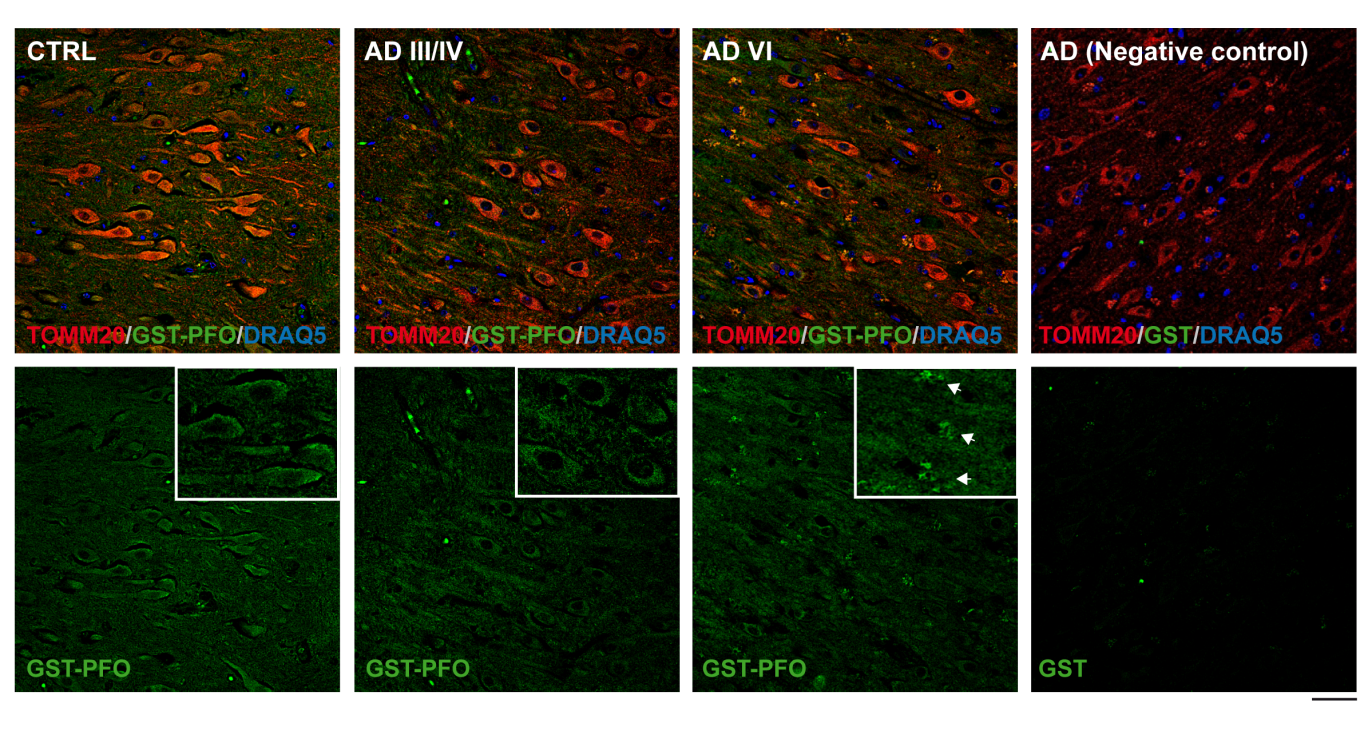


**Figure 15. Differential cholesterol distribution in hippocampal neurons with the progression of neuropathological AD stages.** Hippocampal slices from control (CTRL) subjects and individuals with AD classified into three groups according to their neuropathological hallmarks: CTRL, intermediate AD (AD III-IV) and high AD (AD VI). The sections were incubated with GST-PFO (20 μg/m) for 3 h prior immunolabeling. Shown are representative confocal photomicrograph of double immunofluorescence for GST-PFO (green) and TOMM20 (red). A negative control without GST-PFO incubation was included. Nuclei were counterstained by DRAQ5 (blue). White arrows indicate GST-PFO-immunopositive aggregates. n = 5 individuals per group. Scales bar: 50 μm.


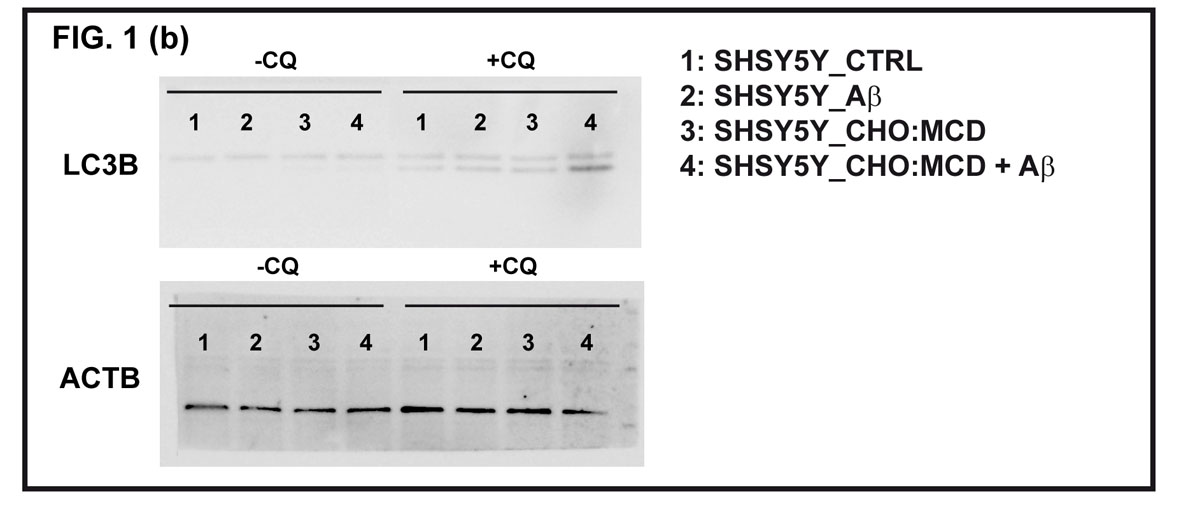


**Figure 16.** Uncropped scans of western blots included in **Figures 1**.


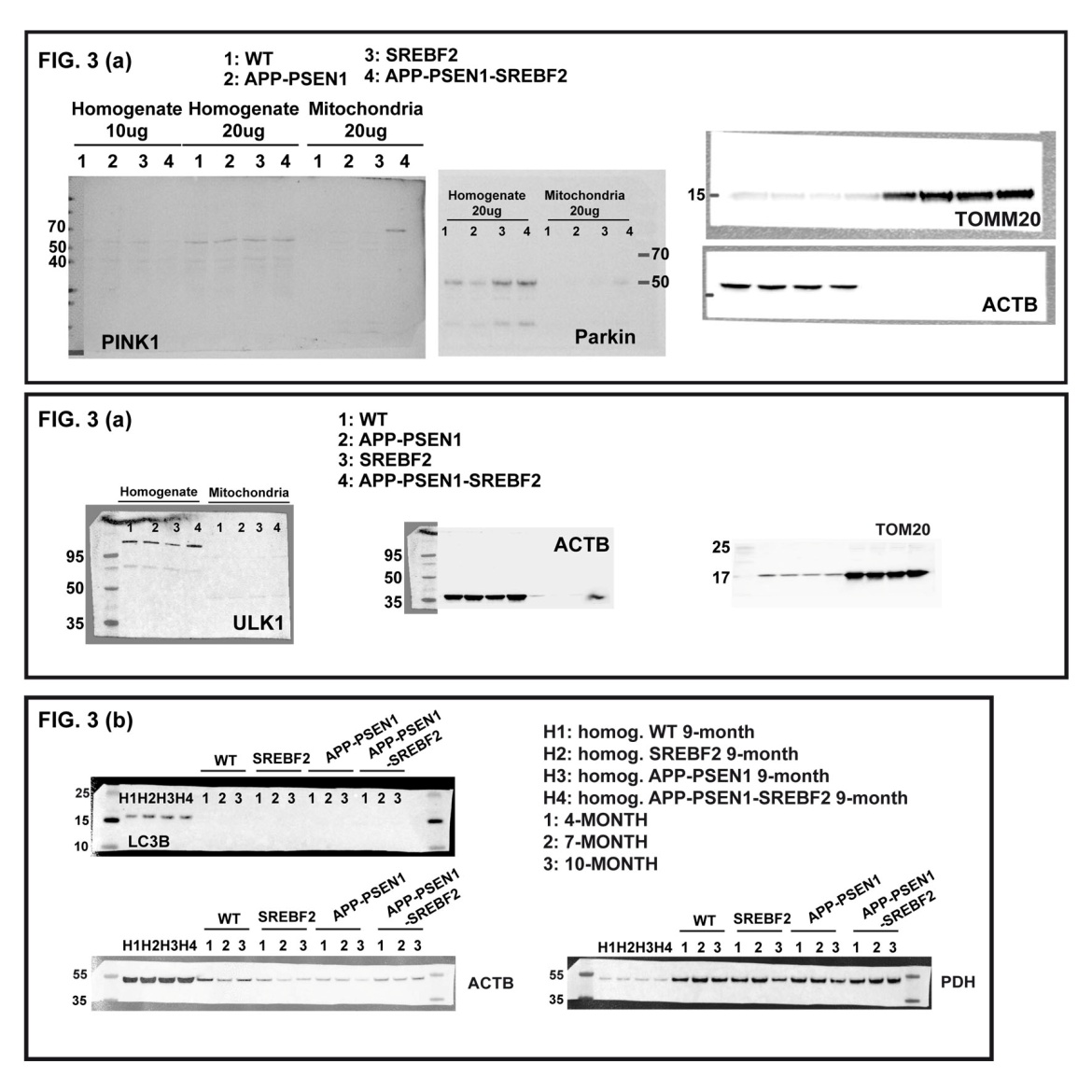
**
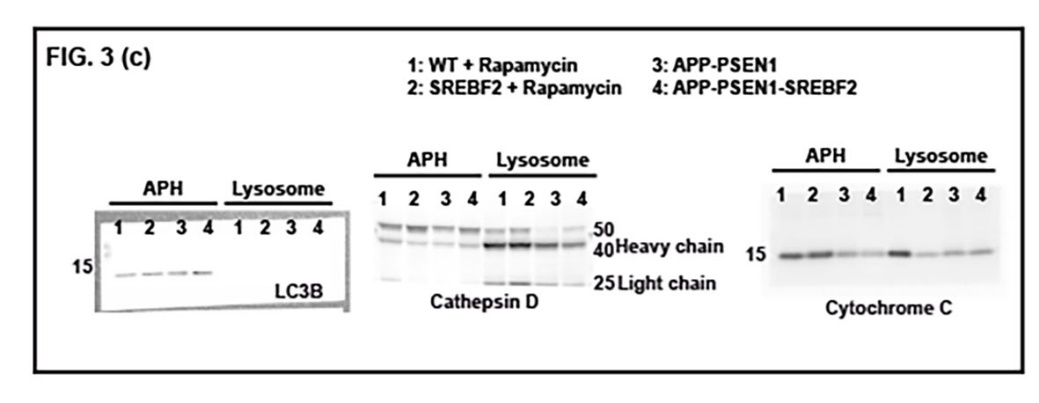
**

**
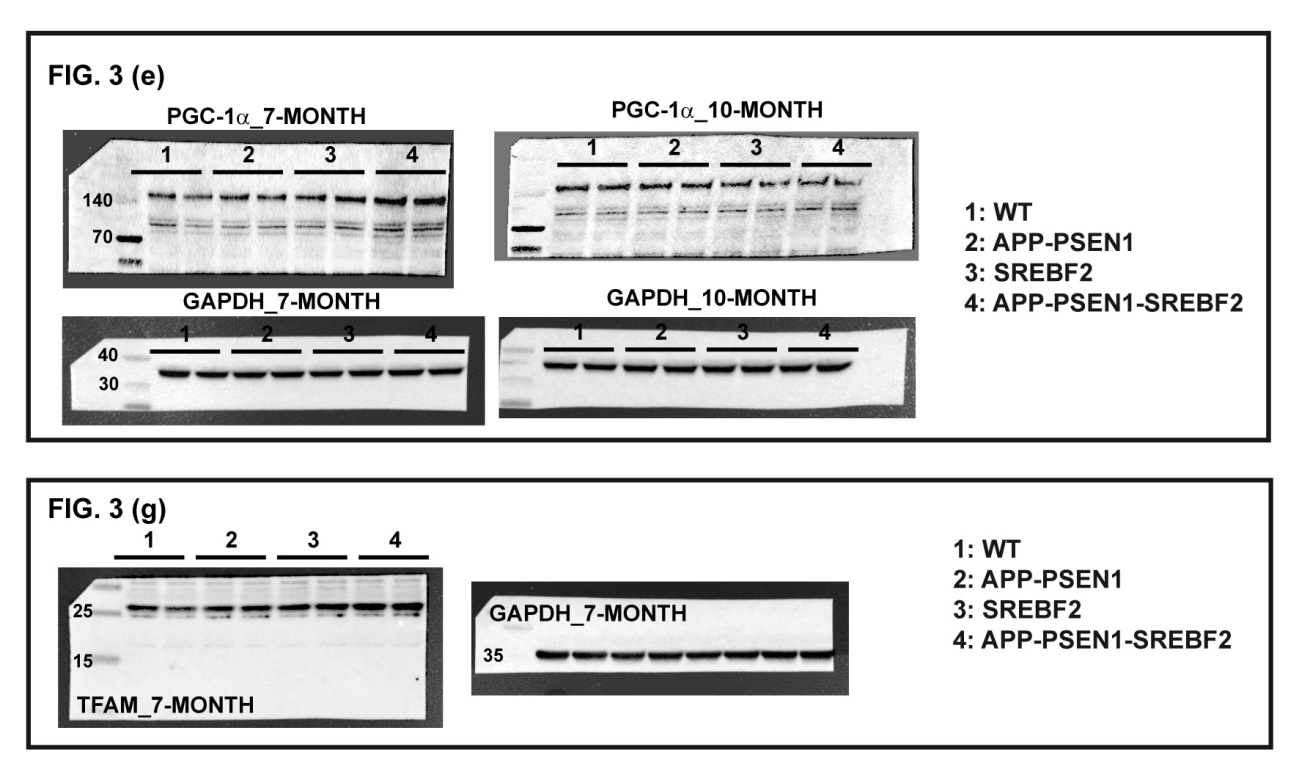
**

**Figure 17.** Uncropped scans of western blots included in **Figures 3**.


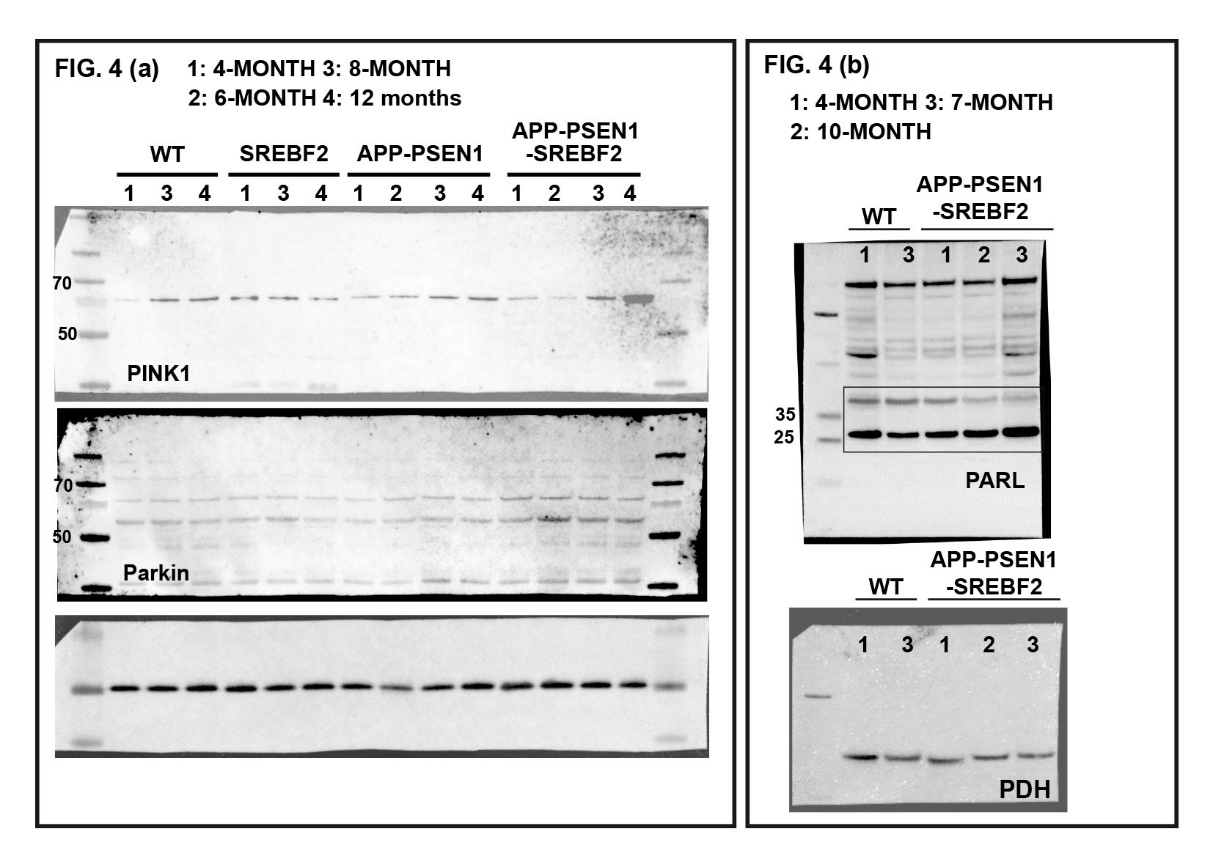


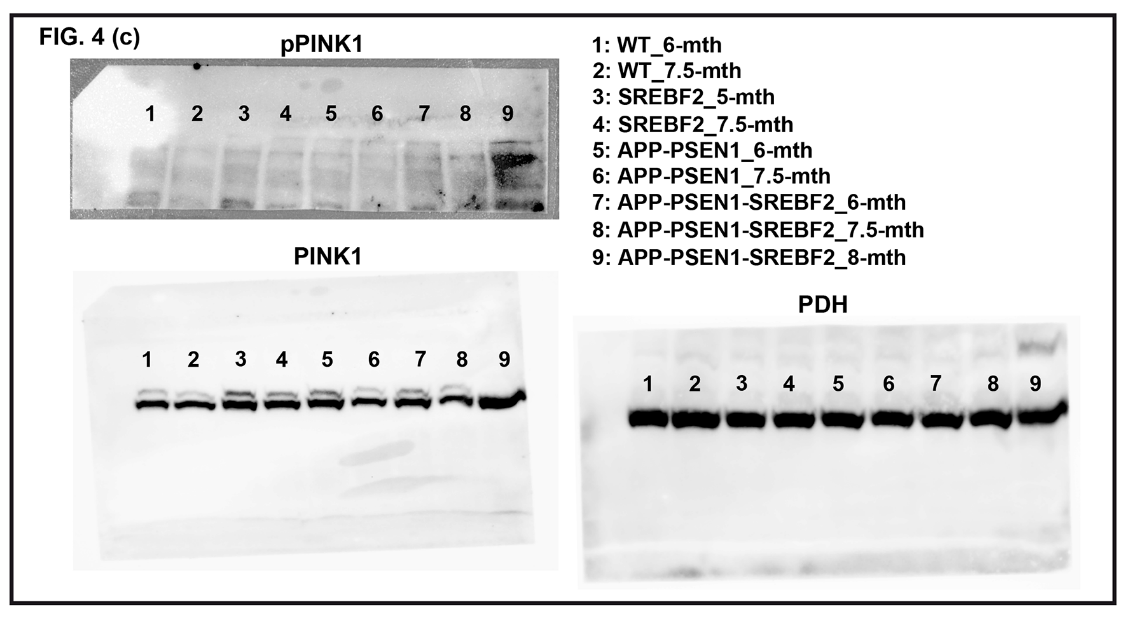


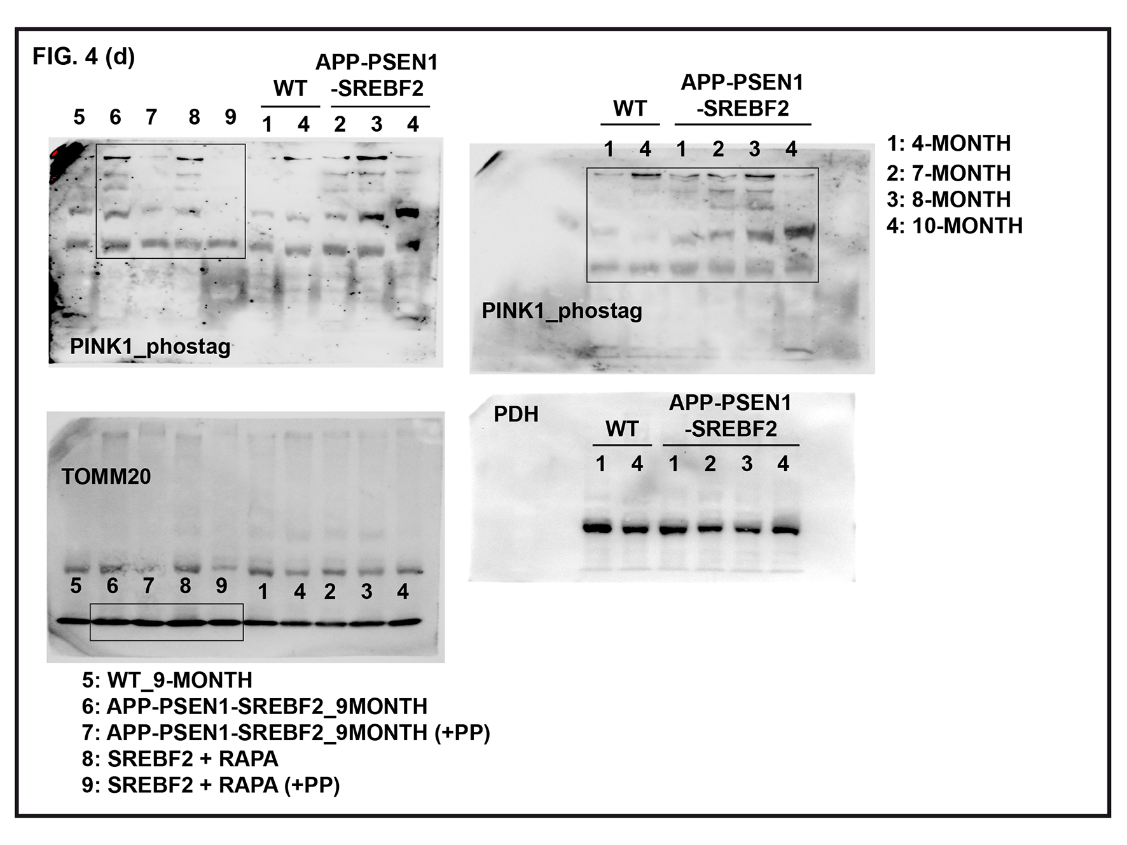


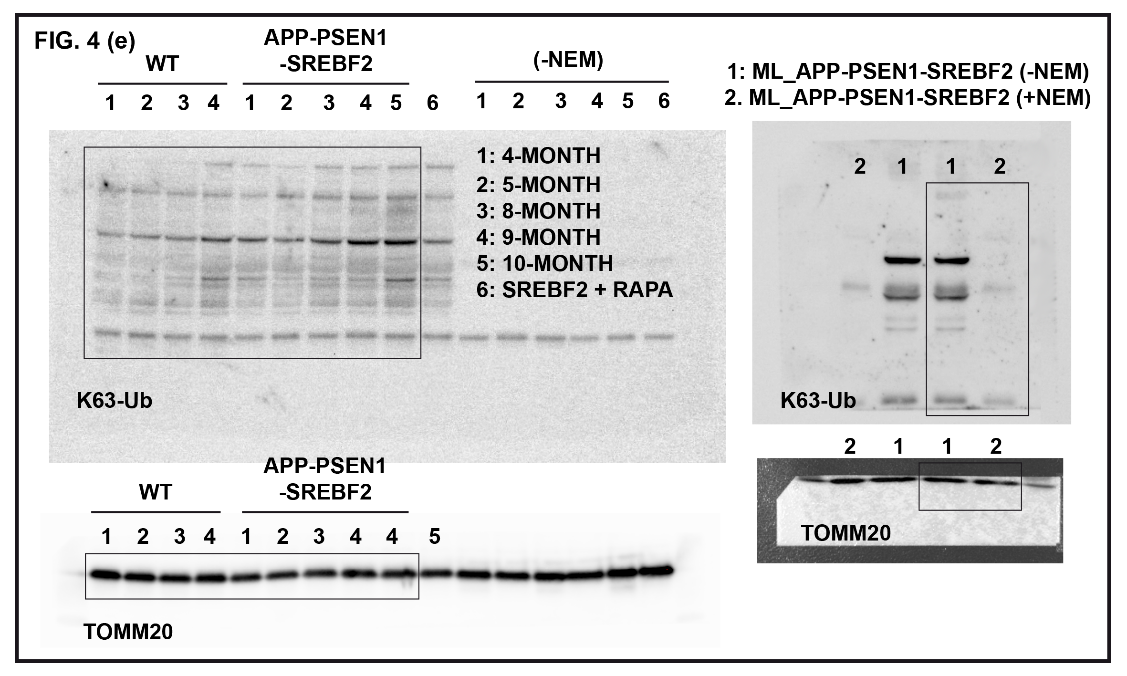


**Figure 18.** Uncropped scans of western blots included in **Figure 4**.


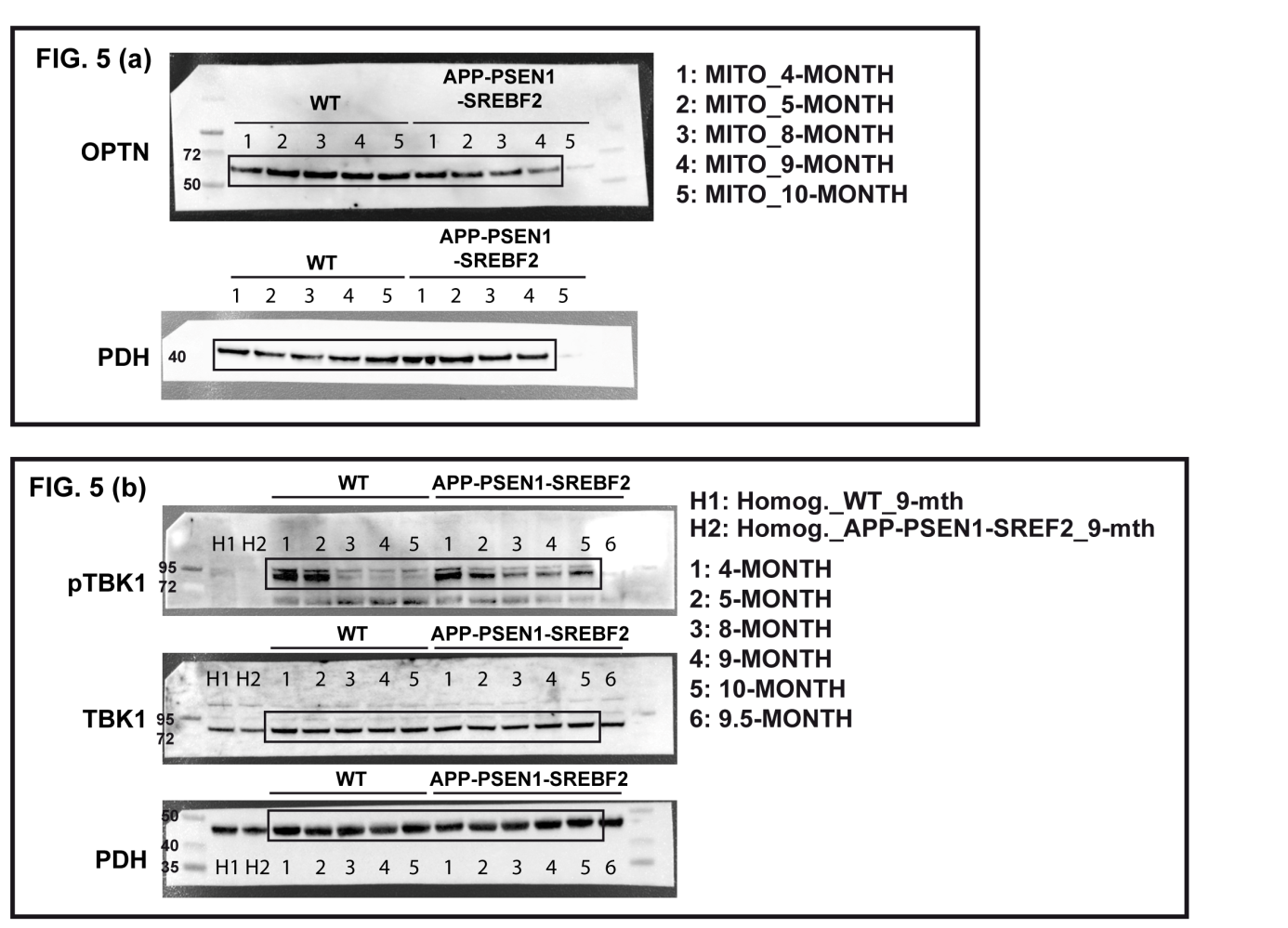


**Figure 19.** Uncropped scans of western blots included in **Figures 5**.
